# National Trends & Disparities in Ischemic Heart Disease & Cardiac Arrhythmias Related Mortality in the United States From 1999 to 2024: A CDC Wonder Analysis

**DOI:** 10.1002/clc.70427

**Published:** 2026-07-27

**Authors:** Muhammad Murtaza, Muhammad Mujtaba Rasool, Sheikh Muhammad Ebtehaj Ali, Muhammad Farhan, Laiba Ilyas, Ahmed Asad Raza, Hammad Amjad, Sumet Kumar, Zoha Khan, Sadia Tahir, Abedin Samadi

**Affiliations:** ^1^ Services Institute of Medical Sciences Lahore Pakistan; ^2^ Allama Iqbal Medical College Lahore Pakistan; ^3^ Abbasi Shaheed Hospital Karachi Pakistan; ^4^ Karachi Medical and Dental College Karachi Pakistan; ^5^ Jinnah Sindh Medical University Karachi Pakistan; ^6^ Shaheed Mohtarma Benazir Bhutto Medical College Lyari Karachi Pakistan; ^7^ POF Hospital Wah Cantt Pakistan; ^8^ Kabul University of Medical Sciences Abu Ali Sina Kabul Afghanistan

**Keywords:** cardiac arrhythmias, cardiovascular epidemiology, CDC WONDER, ischemic heart disease, mortality trends

## Abstract

**Background:**

Ischemic heart disease and cardiac arrhythmias are major contributors to cardiovascular mortality in the United States. Understanding long‐term national trends and demographic disparities is essential for guiding prevention strategies and public health policies.

**Methods:**

A retrospective cross‐sectional analysis was conducted using mortality data from the Centers for Disease Control and Prevention Wide‐ranging Online Data for Epidemiologic Research (CDC WONDER) database from 1999 to 2024. Deaths in which IHD and a cardiac arrhythmia were both recorded were identified using ICD‐10 codes. Crude mortality rates and age‐adjusted mortality rates per 100,000 population were calculated. Temporal trends were assessed using Joinpoint regression to estimate annual percent change (APC) and average annual percent change (AAPC). Mortality patterns were further stratified by sex, race/ethnicity, age group, geographic region, and urbanization status.

**Results:**

A total of 1 885 917 deaths were attributed to IHD and cardiac arrhythmias between 1999 and 2024. The overall AAMR declined from 41.97 in 1999 to 31.75 in 2024. Mortality decreased substantially from 1999 to 2009, stabilized through 2018, and increased sharply during 2018–2021 before declining again through 2024. The cumulative AAMR was 45.09 among males and 23.33 among females. Mortality varied across racial and ethnic groups, geographic regions, urbanization levels, and age groups.

**Conclusion:**

Although overall mortality related to IHD and cardiac arrhythmias has declined over the past two decades, demographic and geographic differences persist. The temporary rise during 2018–2021 coincided with the COVID‐19 pandemic period and underscores the need for sustained prevention strategies and equitable healthcare access.

## Graphical abstract

This graphical abstract summarizes long‐term national trends and demographic disparities in mortality related to co‐occurring ischemic heart disease (IHD) and cardiac arrhythmias in the United States between 1999 and 2024, derived from the Centers for Disease Control and Prevention Wide‐ranging Online Data for Epidemiologic Research (CDC WONDER) database. A total of **1 885 917 deaths** were identified in which both conditions were recorded (ICD‐10 codes I20–I25 for IHD and I44–I49 for cardiac arrhythmias). The overall age‐adjusted mortality rate (AAMR) declined from **41.97 to 31.75 per 100 000** (cumulative AAMR 32.46). Joinpoint regression revealed four distinct phases: a steep decline (1999–2009), a plateau (2009–2018), a pronounced surge to a peak of **36.06 during 2018–2021** coinciding with the COVID‐19 pandemic era, and a subsequent decline through 2024. Substantial disparities persisted across subgroups: mortality was approximately twofold higher in males (AAMR 45.09) than females (23.33); highest among non‐Hispanic White individuals (34.79) and lowest among non‐Hispanic Asian or Pacific Islander individuals (16.44); greater in non‐metropolitan (37.14) than metropolitan areas (31.23); and concentrated among adults aged ≥ 65 years (crude rate 144.27 per 100 000). Vermont, West Virginia, Tennessee, Rhode Island, and Ohio recorded the highest state‐level burden. Although overall mortality has declined over two decades, persistent demographic and geographic inequities underscore the need for sustained, equitable prevention strategies and earlier cardiovascular risk assessment.

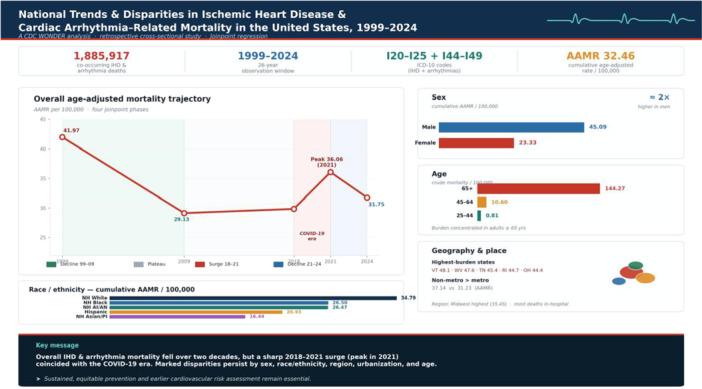

**Abbreviations**
AAMR: age‐adjusted mortality rate
AAPC: average annual percent change
APC: annual percent change
CDC: Centers for Disease Control and Prevention
CI: confidence interval
CMR: crude mortality rate
CVD: cardiovascular disease
ICD: International Classification of Diseases and Related Health Problems
IHD: ischemic heart disease
NH: non‐Hispanic
STROBE: Strengthening the Reporting of Observational Studies in Epidemiology
U.S.: United States
WONDER: Wide‐ranging Online Data for Epidemiologic Research

## 1 Introduction

Cardiovascular diseases (CVDs) remain the leading cause of mortality worldwide and represent a substantial public health burden in the United States. Among cardiovascular conditions, ischemic heart disease (IHD) and cardiac arrhythmias account for a large proportion of cardiovascular morbidity and mortality. IHD, primarily caused by atherosclerotic narrowing of the coronary arteries, leads to myocardial ischemia and infarction, while cardiac arrhythmias arise from abnormalities in cardiac electrical conduction that may precipitate hemodynamic instability and sudden cardiac death. Despite substantial progress in prevention, early detection, and therapeutic interventions, these conditions continue to contribute significantly to global mortality and healthcare expenditure [1, 2, 3]. Epidemiologic analyses consistently demonstrate that IHD remains one of the most common causes of death globally and in the US population, accounting for millions of deaths annually [4].

The pathophysiological relationship between IHD and cardiac arrhythmias is well established. Myocardial ischemia can induce structural and electrical remodeling of cardiac tissue, including myocardial fibrosis, autonomic imbalance, and conduction system abnormalities, thereby increasing susceptibility to malignant ventricular arrhythmias and sudden cardiac death [5, 6]. In patients with coronary artery disease, arrhythmias such as ventricular tachycardia and ventricular fibrillation represent major mechanisms of mortality, particularly following myocardial infarction [7]. Additionally, atrial arrhythmias—including atrial fibrillation—are commonly observed in patients with ischemic or structural heart disease and are associated with increased risks of stroke, heart failure, and mortality [8].

IHD and cardiac arrhythmias are two distinct clinical entities, sharing closely interconnected pathophysiological mechanisms. Moreover, electrical instability due to ischemic injury of myocardial tissue predisposes individuals to atrial and ventricular arrhythmias [9, 10]. Conversely, arrhythmias are frequently reported as an immediate or contributing cause of death in patients with IHDs [7]. Because ischemic and arrhythmic processes are mechanistically coupled and frequently converge in the same terminal event, and because death‐certificate coding cannot reliably separate the underlying ischemic substrate from its arrhythmic mechanism [11], we examined deaths in which both conditions were recorded. This composite reflects a shared pathophysiological cascade rather than two independent processes, and provides a more faithful estimate of the combined population‐level burden than either code considered in isolation.

Several cardiometabolic risk factors contribute to the development and progression of both IHD and cardiac arrhythmias. Hypertension, diabetes mellitus, dyslipidemia, obesity, tobacco use, and sedentary lifestyle collectively drive atherosclerotic vascular disease and myocardial remodeling, thereby increasing the risk of both ischemic events and electrical instability within the myocardium [5, 12]. These shared pathophysiological pathways underscore the importance of evaluating ischemic and arrhythmic cardiovascular conditions together when assessing overall cardiovascular mortality trends.

Over the past several decades, significant advances in cardiovascular prevention and management—including statin therapy, antiplatelet medications, revascularization procedures, and improvements in emergency cardiac care—have contributed to declining mortality from IHD in many high‐income countries. Population‐level analyses using national mortality databases have demonstrated substantial reductions in age‐adjusted mortality rates (AAMRs) from cardiovascular causes during the early 2000s [3, 13]. However, these favorable trends have shown signs of stagnation in recent years, with some studies reporting a slowing decline or even temporary increases in cardiovascular mortality during the late 2010s [14].

Despite these overall improvements, significant disparities persist across demographic and socioeconomic subgroups within the United States. Age remains one of the strongest determinants of cardiovascular mortality, with individuals aged 65 years and older experiencing markedly higher mortality rates due to cumulative exposure to cardiometabolic risk factors and age‐related vascular changes. Additionally, sex‐based differences in cardiovascular mortality have been widely reported, with males demonstrating higher mortality rates compared with females [15]. Racial and ethnic disparities further highlight persistent inequities in cardiovascular health outcomes, influenced by differences in socioeconomic status, healthcare access, and the distribution of cardiovascular risk factors among populations [16].

Geographic and urbanization‐related differences further contribute to heterogeneity in cardiovascular mortality patterns across the United States. Previous nationwide studies have reported higher mortality rates from IHD and cardiac arrhythmias in certain regions, particularly in rural and non‐metropolitan areas compared with metropolitan regions. These differences are frequently attributed to variations in healthcare infrastructure, availability of specialized cardiovascular services, and regional differences in lifestyle‐related risk factors such as obesity and smoking [17, 18].

Beyond broad regional comparisons, substantial state‐level heterogeneity in IHD mortality has been documented; Essa et al. described marked variation in age‐adjusted IHD mortality and its temporal trends across individual US states between 1999 and 2019 [19]. However, state‐level trends for the combined burden of ischemic and arrhythmic mortality and whether high‐mortality states are improving or worsening over time remain uncharacterized. Analyzing mortality at the state level can therefore distinguish states with a high but declining burden from those with persistently high or rising mortality, a distinction that regional aggregation obscures.

Recent epidemiological evidence has also highlighted emerging challenges in cardiovascular mortality trends associated with major healthcare disruptions. The COVID‐19 pandemic was associated with substantial changes in healthcare utilization, delays in emergency cardiac care, and increased inflammatory and thrombotic states in affected patients. These factors may have contributed to the observed rise in cardiovascular mortality during the pandemic period in several national analyses [20, 21, 22].

Although numerous studies have evaluated cardiovascular mortality trends in the United States, comprehensive long‐term assessments specifically examining mortality patterns related to both IHD and cardiac arrhythmias across demographic and geographic subgroups remain limited. Many existing investigations focus on single cardiovascular conditions or shorter observational periods, restricting the ability to fully understand evolving national patterns in cardiovascular mortality [14, 23]. Therefore, a comprehensive evaluation of long‐term mortality trends across multiple population strata is essential to better characterize the epidemiological burden of these conditions.

Most directly relevant, Saad et al. used CDC WONDER to examine mortality among US adults with coexisting coronary artery disease and atrial fibrillation from 1999 to 2020, applying the same IHD code range (I20–I25), direct age standardization, and Joinpoint regression, with a comparable demographic and geographic stratification [24]. Both that study and the present analysis examine deaths in which ischemic and arrhythmic disease co‐occur; three features distinguish the current work. First, Saad et al. restricted the arrhythmic component to atrial fibrillation (I48), whereas we include the full spectrum of cardiac arrhythmias (I44, I45, I47, I48, I49), capturing the ventricular and conduction arrhythmias that constitute the principal mechanism of sudden cardiac death in IHD rather than atrial fibrillation alone. Second, by extending observation through 2024, the present study characterizes the entire pandemic‐era trajectory: the 2018–2021 rise, the 2021 peak, and the subsequent decline through 2024 whereas a window ending in 2020 captures only the initial upslope. Third, it adds age‐stratified and place‐of‐death analyses that have not been examined previously. Together, these provide a broader and more current characterization of co‐occurring ischemic and arrhythmic mortality at the population level.

Accordingly, the present study utilizes mortality data from the Centers for Disease Control and Prevention Wide‐Ranging Online Data for Epidemiologic Research (CDC WONDER) database to evaluate long‐term mortality trends associated with IHD and cardiac arrhythmias in the U.S. from 1999 to 2024. This study aims to examine temporal changes in mortality rates and identify disparities across demographic, geographic, and urbanization subgroups, thereby providing updated epidemiological insights to inform future cardiovascular prevention strategies and public health policies.

## 2 Methods

### 2.1 Study Setting

In this retrospective cross‐sectional study, death certificate data from 1999 to 2024 were retrieved and analyzed from the Centers for Disease Control and Prevention (CDC) Wide‐ranging Online Data for Epidemiologic Research (WONDER) database, which is a publicly available online database that contains public health data, including mortality data, since the year 1999 [25]. A death was included only if both IHD and cardiac arrhythmia were recorded on the same death certificate either as an underlying or contributing cause of death. The composite outcome therefore reflects co‐occurring ischemic and arrhythmic mortality, not either condition alone. Data extraction was done using International Classification of Diseases and Related Health Problems‐10th Revision (ICD‐10) codes: I20–25 for IHD and I44.0–I44.7, I45.0–I45.6, I45.8, I45.9, I47.0, I47.1, I47.2, I47.9, I48, I 49.0–I49.5, I49.8, I49.9 for cardiac arrhythmias. These same ICD‐10 codes have been used previously to identify IHD and cardiac arrhythmias in other studies [26, 27]. Since the study used a publicly available deidentified dataset, institutional review board approval was not necessary. The study is based on Strengthening the Reporting of Observational Studies in Epidemiology (STROBE) guidelines [28].

### 2.2 Data Extraction

The dataset was analyzed by population, year, gender, race/ethnicity, age group, geographic region, state, place of death, and metropolitan/non‐metropolitan classification. Racial and ethnic categories were defined as non‐Hispanic (NH) White, NH Black or African American, NH American Indian or Alaska Native, NH Asian or Pacific Islander, and Hispanic or Latino. Age groups were categorized as 25–44, 45–64, and 65+ years. The place of death was classified into six categories: medical facilities (inpatient, outpatient/emergency), hospice facilities, the decedent's home, nursing homes/long‐term care facilities, other locations, and unknown. According to the 2013 US Census Bureau Classifications, the study population was categorized into metropolitan and non‐metropolitan areas, and regions were defined as Northeast, Midwest, South, and West [29].

### 2.3 Statistical Analysis

Crude mortality rates (CMRs) and AAMRs per 100 000 individuals with 95% confidence intervals (CIs) were calculated for deaths related to IHD and cardiac arrhythmias. CMRs were determined by dividing the number of IHD and cardiac arrhythmias‐related deaths by the corresponding US population of that year. AAMRs and 95% CIs were obtained from CDC WONDER, which applies direct standardization to the 2000 US standard population [30]. AAMRs were used to analyze the mortality trends based upon year, overall population, sex, and race/ethnicity, whereas CMRs were used to investigate the mortality trends by age. For geographical variations, we also cross‐examined the AAMRs across different regions, states, and metropolitan versus non‐metropolitan status. Joinpoint trend analysis was performed to determine the average annual percent change (AAPC) in AAMR trends over the years. Joinpoint regression analysis was conducted through the Joinpoint Regression Program (Version 5.3.0, November 2024; Statistical Research and Applications Branch, National Cancer Institute) to calculate annual percent changes (APCs) and AAPCs in AAMR trends over the years, with a 95% CI [31].

This method identifies significant changes in AAMR over time by fitting a log‐linear regression model over temporal variation. APCs were considered as increasing or decreasing and written as “positive“ or “negative,” respectively, if the slope describing the change in mortality was significantly different from zero using a two‐tailed *t*‐test. The value of APC was considered statistically significant if its *p* value was less than 0.05 and its 95% CI excluded zero.

## 3 Results

### 3.1 Overall

IHD and cardiac arrhythmias contributed to 1 885 917 deaths in the United States from 1999 to 2024, underscoring a substantial and persistent mortality burden (Table 1; Supporting Information S1: Tables 1 and 2). Over this period, the overall AAMR declined from 41.97 to 31.75, with a cumulative AAMR of 32.46 (95% CI: 32.22–32.70) (Supporting Information S1: Table 3).

**Table 1:** Ischemic heart disease and Cardiac Arrhythmia‐related mortality in the United States from 1999–2024.

Variable	Ischemic heart disease and cardiac arrhythmia related total deaths	AAMRs/CMRs (95% CI) per 100 000
*Overall*	1 885 917 (100%)	32.46 (32.22–32.70)
*Sex* a		
Male	1 069 904 (56.74%)	45.09 (44.65–45.54)
Female	816 013 (43.26%)	23.33 (23.07–23.59)
*Census region* a		
Northeast	365 783 (19.40%)	31.62 (31.10–32.15)
Midwest	465 161 (24.66%)	35.45 (34.93–35.97)
South	659 063 (34.95%)	31.27 (30.88–31.67)
West	395 910 (20.99%)	31.93 (31.42–32.45)
*Race* a		
NH White	1 616 437 (85.71%)	34.79 (34.52–35.07)
Hispanic or Latino	85 611 (4.54%)	20.93 (20.16–21.70)
NH Black or African American	135 124 (7.16%)	26.50 (25.76–27.25)
NH Asian or Pacific Islander	35,785 (1.90%)	16.44 (15.49–17.39)
NH American Indian or Alaska Native	7970 (0.42%)	26.47 (23.28–29.69)
*Urbanization* a		
Metropolitan	1 208 578 (64.08%)	31.23 (30.96–31.49)
Nonmetropolitan	311 604 (16.52%)	37.14 (36.52–37.76)
*Age* b		
25–44 years	17 812 (0.94%)	0.81 (0.75–0.87)
45–64 years	212 231 (11.25%)	10.60 (10.37–10.83)
65+ years	1 655 874 (87.80%)	144.27 (143.14–145.40)
*Place of death* c		
Decedent's home	478 091 (25.35%)	—
Inpatients (medical facility)	649 978 (34.46%)	—
Outpatients (medical facility)	215 046 (11.40%)	—
Nursing homes	394 030 (20.89%)	—
Hospice	58 760 (3.12%)	—
Others	68 216 (3.62%)	—
Dead on arrival	17 683 (0.94%)	—
Unknown	2861 (0.15%)	—

Abbreviations: AAMRs, age adjusted mortality rates; CMRs, crude mortality rates; CI, confidence interval; IHD, ischemic heart disease; NH, non‐Hispanic.

^a^ Age‐adjusted mortality rates (AAMRs) used.

^b^ Crude mortality rates (CMRs) used for age groups.

^c^ AAMRs not applicable for place of death.

**Figure 1:**
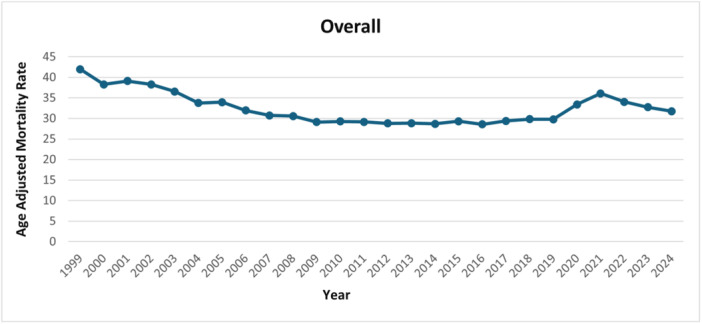
Ischemic heart disease and cardiac arrhythmia‐related mortality among adults in the United States, 1999–2024.

Temporal trend analysis demonstrated an initial decline from 41.97 in 1999 to 29.13 in 2009 (APC: –3.49; 95% CI: –4.00 to –3.09; *p* < 0.000001), followed by relative stabilization through 2018. A pronounced increase occurred during 2018–2021, with the AAMR rising from 29.84 to a peak of 36.06 (APC: 7.08; 95% CI: 4.69 to 8.32; *p* < 0.000001). Thereafter, mortality rates declined again from 2021 to 2024, reaching 31.75 (APC: –3.90; 95% CI: –5.49 to –2.52; *p* < 0.000001) (Figure 1) (Supporting Information S1: Table 4).

### 3.2 Gender

From 1999 to 2024, the male population accounted for 1 069 904 deaths (56.7%), whereas 816 013 deaths (43.3%) occurred among females (Table 1). The cumulative AAMR was 45.09 (95% CI: 44.65–45.54) among males and 23.33 (95% CI: 23.07–23.59) among females (Supporting Information S1: Table 3). In both sexes, mortality declined, stabilized, and rose transiently during 2018–2021.

**Figure 2:**
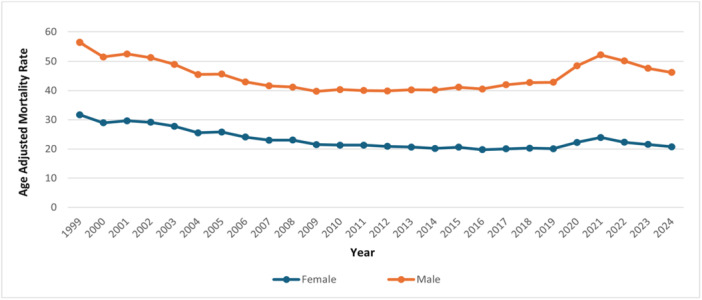
Ischemic heart disease and cardiac arrhythmia‐related mortality among adults stratified by sex in the United States, 1999–2024.

Among females, the AAMR declined from 31.72 in 1999 to 20.77 in 2024, with a cumulative AAMR of 23.33 (95% CI: 23.07–23.59) (Supporting Information S1: Table 3). The AAMR decreased from 31.72 in 1999 to 21.53 in 2009 (APC = –3.69; 95% CI: –4.42 to –3.24; *p* < 0.000001), followed by a continued but slower decrease to 20.31 in 2018 (APC = –1.09; 95% CI: –1.86 to –0.30; *p* = 0.013). A sharp increase was observed during 2018–2021, with AAMR rising from 20.31 to 23.95 (APC = 6.21; 95% CI: 3.36 to 7.66; *p* < 0.000001), after which rates again declined to 20.77 in 2024 (APC = –4.27; 95% CI: –6.73 to –2.62; *p* < 0.000001) (Figure 2) (Supporting Information S1: Table 4). Overall, female rates demonstrated a long‐term downward trend with a temporary surge during 2018–2021.

Among males, the AAMR decreased from 56.50 in 1999 to 46.21 in 2024, with a cumulative AAMR of 45.09 (95% CI: 44.65–45.54) (Supporting Information S1: Table 3). Rates declined from 56.50 in 1999 to 39.75 in 2009 (APC = –3.35; 95% CI: –3.88 to –2.94; *p* < 0.000001) followed by a stabilization period till 2018. Relatively high trend observed during 2018–2021, with AAMR rising from 42.77 to 52.21 (APC = 7.61; 95% CI: 5.48 to 8.82; *p* < 0.000001), followed by a decline to 46.21 in 2024 (APC = –3.85; 95% CI: –5.32 to –2.50; *p* < 0.000001) (Figure 2) (Supporting Information S1: Table 4).

### 3.3 Race

Throughout the study period, AAMRs varied across racial and ethnic groups. Cumulative AAMRs by group were NH White 34.79 (95% CI: 34.52 to 35.07), NH Black or African American 26.50 (95% CI: 25.76 to 27.25), NH American Indian or Alaska Native 26.47 (95% CI: 23.28 to 29.69), Hispanic or Latino 20.93 (95% CI: 20.16 to 21.70), and NH Asian or Pacific Islander 16.44 (95% CI: 15.49 to 17.39) (Table 1).

**Figure 3:**
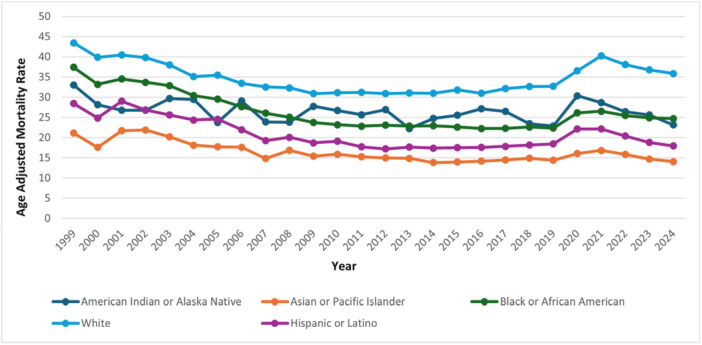
Ischemic heart disease and cardiac arrhythmia related mortality among adults stratified by race/ethnicity in the United States, 1999–2024.

Among NH White individuals, the AAMR declined from 43.47 in 1999 to 35.87 in 2024 (Supporting Information S1: Table 5). Rates dropped to 30.90 in 2009 (APC = –3.24; 95% CI: –3.75 to –2.83; *p* < 0.000001), plateaued during 2009–2018, increased from 32.67 to 40.23 during 2018–2021 (APC = 7.56; 95% CI: 5.07 to 8.84; *p* < 0.000001), and subsequently declined to 35.87 in 2024 (APC = –3.29; 95% CI: –4.98 to –1.86; *p* < 0.000001) (Figure 3) (Supporting Information S1: Table 4).

The AAMR among NH Black individuals decreased from 37.42 in 1999 to 24.72 in 2024 (Supporting Information S1: Table 5). Rates declined sharply from 37.42 in 1999 to 23.20 in 2010 (APC = –4.22; 95% CI: –5.10 to –3.71; *p* = 0.001), then stabilized during 2010–2018. A period of steep incline between 2018 and 2021 was seen where the AAMR rose from 22.63 to 26.53 (APC = 6.19; 95% CI: 2.91 to 8.04; *p* = 0.006), and finally a decline to 24.72 in 2024 was noted (APC = –2.72; 95% CI: –6.50 to –0.67; *p* = 0.02) (Figure 3) (Supporting Information S1: Table 4).

Among NH American or Alaska Native, the AAMR decreased from 33.03 in 1999 to 23.18 in 2024 (Supporting Information S1: Table 5). However, the overall trend exhibited a non‐significant decline across the study period (APC = –0.39; 95% CI: –0.91 to 0.22; *p* = 0.23), with no identified joinpoints. (Figure 3) (Supporting Information S1: Table 4).

For Hispanic or Latino individuals, the AAMR declined from 28.48 in 1999 to 17.94 in 2024 (Supporting Information S1: Table 5). Initially the rates decreased from 28.48 in 1999 to 17.41 in 2014 (APC = –3.76; 95% CI: –4.59 to –3.03; *p* = 0.0008), followed by an increase to 22.16 in 2021 (APC = 4.17; 95% CI: 2.53 to 8.94; *p* = 0.003), and then declined markedly from 2021 to 2024 (APC = –6.30; 95% CI: –12.41 to –2.17; *p* = 0.003) (Figure 3) (Supporting Information S1: Table 4).

Among NH Asian or Pacific Islander, the AAMR decreased from 21.13 in 1999 to 14.09 in 2024 (Supporting Information S1: Table 5). Between 1999 and 2015 a noticeable decline from 21.13 to 13.94 was observed (APC = –2.76; 95% CI: –3.53 to –2.11; *p* = 0.007), followed by a rise to 16.83 in 2021 (APC = 3.19; 95% CI: 1.35 to 7.94; *p* = 0.02), and subsequent decline to 14.09 in 2024 (APC = –5.04; 95% CI: –10.65 to –1.59; *p* = 0.02) (Figure 3) (Supporting Information S1: Table 4).

### 3.4 Census Regions

**Figure 4:**
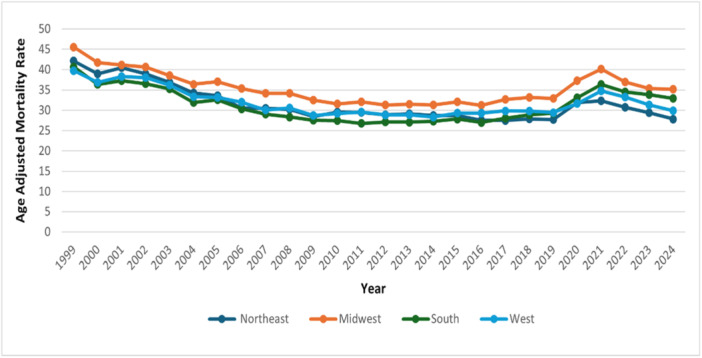
Ischemic heart disease and cardiac arrhythmia‐related mortality among adults stratified by census regions in the United States, 1999–2024.

In the Midwest, the AAMR declined from 45.53 in 1999 to 35.15 in 2024, with a cumulative AAMR of 35.45 (95% CI: 34.93 to 35.97) (Supporting Information S1: Table 6). Rates prominently decreased from 1999 to 2010 (APC = –3.02; 95% CI: –4.12 to –2.51; *p* = 0.004), before displaying a period of stability till 2018. Between 2018 and 2021, the AAMR rose significantly from 33.18 to 40.15 (APC = 6.90; 95% CI: 3.52 to 8.64; *p* < 0.000001). This was finally followed by a decline to 35.15 in 2024 (APC = –4.24; 95% CI: –7.40 to –2.42; *p* = 0.0008) (Figure 4) (Supporting Information S1: Table 4).

In the West, the AAMR decreased from 39.75 in 1999 to 29.86 in 2024, with a cumulative AAMR of 31.93 (95% CI: 31.42–32.45) (Supporting Information S1: Table 6). After an initial decline from 39.75 in 1999 to 28.73 in 2009 (APC = –3.15; 95% CI: –4.33 to –2.57; *p* = 0.003), the AAMR remained relatively constant through 2018. This was followed by a prominent increase from 29.82 to 34.75 during 2018–2021 (APC = 5.44; 95% CI: 2.38 to 7.02; *p* < 0.000001), and then finally a sharp decline to 29.86 in 2024 was seen (APC = −4.22; 95% CI: –7.29 to –2.31; *p* < 0.000001) (Figure 4) (Supporting Information S1: Table 4).

In the Northeast, the AAMR declined from 42.15 in 1999 to 27.83 in 2024, with a cumulative AAMR of 31.62 (95% CI: 31.10–32.15) (Supporting Information S1: Table 6). The AAMR fell sharply from 42.15 in 1999 to 30.50 in 2007 (APC = –4.18; 95% CI: –5.21 to –3.45; *p* < 0.000001), followed by a slower decline to 27.85 in 2018 (APC = –0.92; 95% CI: –1.57 to –0.31; *p* = 0.01). An increase was observed during 2018–2021, with AAMR rising from 27.85 to 32.30 (APC = 5.89; 95% CI: 2.69 to 7.47; *p* < 0.000001), before declining again through 2024 (APC = –5.13; 95% CI: –8.21 to –3.22; *p* < 0.000001) (Figure 4) (Supporting Information S1: Table 4).

For the South, AAMR decreased from 40.68 in 1999 to 32.94 in 2024, with a cumulative AAMR of 31.27 (95% CI: 30.88–31.67) (Supporting Information S1: Table 6). The AAMR dropped from 40.68 in 1999 to 27.51 in 2009 (APC = –3.82; 95% CI: –4.47 to –3.33; *p* < 0.000001). After being plateaued till 2018, the AAMR rose sharply from 28.86 to 36.35 between 2018 and 2021 (APC = 8.87; 95% CI: 6.03 to 10.33; *p* < 0.000001), representing the largest regional increase, before declining to 32.94 in 2024 (APC = −2.91; 95% CI: –4.90 to –1.33; *p* = 0.002) (Figure 4) (Supporting Information S1: Table 4).

### 3.5 Urbanization

**Figure 5:**
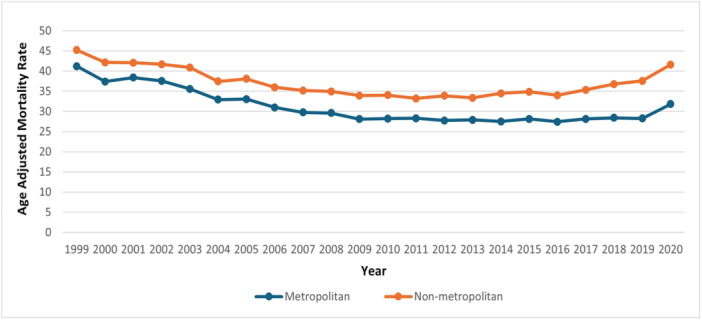
Ischemic heart disease and cardiac arrhythmia related mortality among adults stratified by urbanization in the United States, 1999–2020.

The trends in AAMRs showed distinct patterns between metropolitan and non‐metropolitan areas from 1999 to 2020. In metropolitan areas, the AAMR declined from 41.22 in 1999 to 31.85 in 2020, with a cumulative AAMR of 31.23 (95% CI: 30.96–31.49) (Supporting Information S1: Table 7). Rates decreased from 41.22 in 1999 to 28.12 in 2009 (APC = –3.64; 95% CI: –4.28 to –3.17; *p* = 0.0004), followed by relative stability from 2009 to 2018. A sharp increase was observed during 2018–2020 with AAMR rising from 28.44 to 31.85 (APC = 6.09; 95% CI: 2.05 to 8.25; *p* < 0.000001) (Figure 5) (Supporting Information S1: Table 4).

For non‐metropolitan areas, the AAMR decreased from 45.24 in 1999 to 41.64 in 2020, with a cumulative AAMR of 37.14 (95% CI: 36.52–37.76) (Supporting Information S1: Table 7). The AAMR declined sharply from 45.24 in 1999 to 33.93 in 2009 (APC = –2.82; 95% CI: –3.56 to –2.35; *p* = 0.0004), followed by a phase of stabilization from 2009 to 2017, and then increased markedly from 2017 to 2020, with AAMR rising from 35.34 to 41.64. (APC = 5.70; 95% CI: 3.28 to 9.08; *p* < 0.000001) (Figure 5) (Supporting Information S1: Table 4).

### 3.6 States

**Figure 6:**
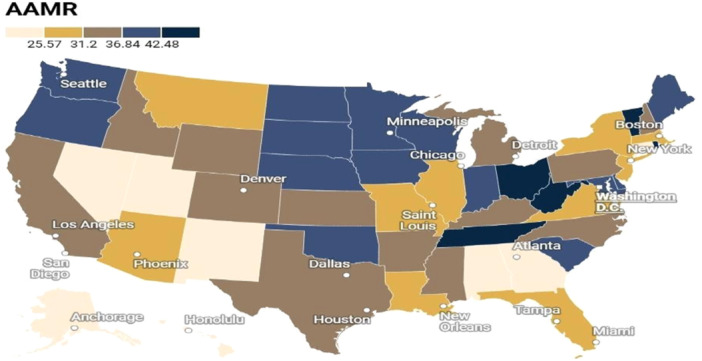
Ischemic heart disease and cardiac arrhythmia‐related mortality among adults stratified by states in the United States, 1999–2024.

State‐level mortality varied considerably across the United States. The five states with the highest cumulative AAMRs were Vermont, West Virginia, Tennessee, Rhode Island, and Ohio. Vermont had the highest cumulative AAMR at 48.12 per 100 000 (95% CI: 46.11 to 50.21), followed by West Virginia at 47.61 (95% CI: 46.41 to 48.85), Tennessee at 45.39 (95% CI: 44.73 to 46.07), Rhode Island at 44.70 (95% CI: 43.21 to 46.24), and Ohio at 44.38 (95% CI: 43.91 to 44.86) (Figure 6) (Supporting Information S1: Table 8).

In Vermont, the AAMR decreased significantly from 1999 to 2004 (APC = −5.32; 95% CI: −14.92 to −0.85; *p* = 0.028), followed by a nonsignificant decline from 2004 to 2024 (APC = −0.57; 95% CI: −5.05 to 4.31; *p* = 0.521). Overall, Vermont demonstrated a significant long‐term decline, with an AAPC of −1.54 (95% CI: −2.18 to −0.61; *p* = 0.002) (Supporting Information S1: Table 4).

The AAMR of West Virginia decreased from 59.55 in 1999 to 48.29 in 2024; however, this decline was not significant (AAPC = −0.50; 95% CI: −1.04 to 0.03; *p* = 0.062) (Supporting Information S1: Table 4). In Tennessee, the AAMR decreased from 52.59 in 1999 to 44.53 in 2024, demonstrating an overall significant decline of 0.49% (95% CI: −0.70 to −0.28; *p* < 0.001) (Supporting Information S1: Table 4).

### 3.7 Age

**Figure 7:**
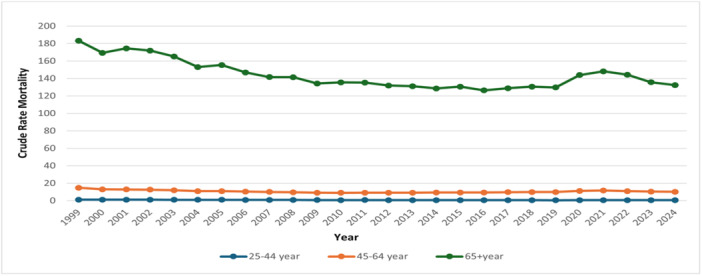
Ischemic heart disease and cardiac arrhythmia related mortality among adults stratified by age groups in the United States, 1999–2024.

Mortality differed across age groups from 1999 to 2024. Deaths totaled 17 812 in those aged 25–44, 212 231 in those aged 45–64, and 1 655 874 in those aged 65 and older. The corresponding cumulative CMRs were 0.81, 10.60, and 144.27 per 100 000, respectively. (Table 1) (Figure 7).

### 3.8 Place of Death

**Figure 8:**
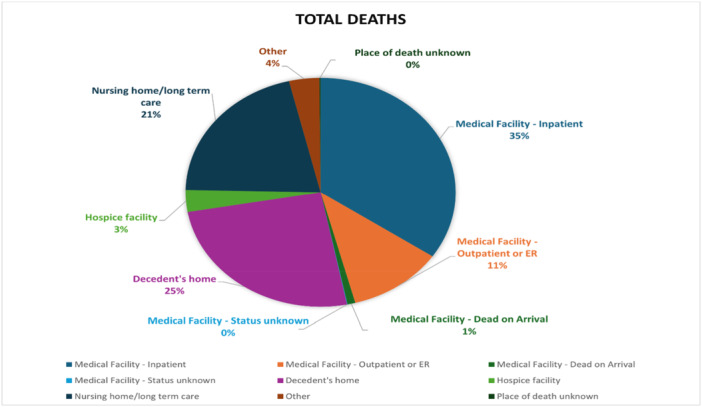
Ischemic heart disease and cardiac arrhythmia‐related mortality among adults stratified by place of death in the United States, 1999–2024.

The distribution of deaths by place reveals that the highest number occurred in medical facilities as inpatient care (649 978 deaths). A notable portion of individuals also passed away at home (478 091), followed by nursing homes or long‐term care facilities (394 030). Deaths in medical facilities as outpatient or emergency room settings reached 215 046, while deaths reported in other settings were 68 216. Smaller numbers were reported in hospice facilities (58 760), medical facilities where the individual was dead on arrival (17 683), and cases where the place of death was unknown (2861) or the status was unspecified (1252) (Table 1) (Figure 8).

Over the study period, notable shifts in the distribution of deaths were observed. Deaths occurring at home more than doubled, increasing from 13 317 in 1999 to 29 266 in 2024. Similarly, hospice facility deaths increased substantially from virtually none in 1999 to 5555 in 2024. In contrast, inpatient medical facility deaths declined from 29 906 in 1999 to 27 499 in 2024 despite population growth and increasing overall mortality burden. Deaths occurring in nursing homes and long‐term care facilities remained relatively stable, ranging from 14 695 to 16 190 annually during most years of the study period (Supporting Information S1: Table 9).

## 4 Discussion

This nationwide analysis of mortality trend related to IHD and cardiac arrhythmias in the US population emphasizes the consistent burden of cardiovascular mortality with geographic and demographic differences. Over the full study period, the overall AAMR declined, reflecting sustained progress in cardiovascular prevention and treatment; however, this decline was not uniform over time. A steep early reduction was followed by a slowing and plateauing of the decline, after which mortality rose from 2018 to a peak in 2021 before declining again through 2024. Notably, the onset of this increase preceded the COVID‐19 pandemic. This pre‐pandemic inflection is consistent with the deceleration and, in some population subgroups, early reversal of the long‐term decline in US cardiovascular mortality that has been documented since the early 2010s [32]. Plausible contributors include plateauing gains in the management of cardiovascular risk factors, the rising prevalence of obesity and metabolic syndrome, and evolving statin utilization patterns [33]; the cardiovascular consequences of the opioid epidemic, evolving ICD‐10 coding practices, and greater recognition of co‐occurring cardiovascular conditions may have further contributed to the observed inflection. The more pronounced rise between 2018 and 2021 encompassed the early COVID‐19 pandemic, a period for which prior analyses have described disruptions in cardiovascular healthcare utilization [34]; because of the cross‐sectional design, however, this temporal overlap cannot be interpreted as a causal or explanatory relationship. Taken together, these observations describe long‐term progress in reducing cardiovascular mortality alongside more recent challenges that warrant continued surveillance.

The initial decline in mortality rate observed between 1999 and 2009 reflects the positive advancements in cardiovascular care, widespread use of antiplatelet therapy and statins, improved revascularization techniques, and emergency cardiac care systems [35, 36]. These favorable trends were contributed by early diagnosis of coronary artery diseases and rising awareness of cardiovascular risk factors throughout the United States [37]. Most importantly, these findings were consistent with previous nationwide studies of the early 2000s [38, 39].

In this analysis, mortality differed by sex. Major contributors to this rising trend in male subgroups were a high prevalence of cardiovascular risk factors such as metabolic syndrome and a history of chronic smoking [40]. Most importantly, biological factors such as hormonal influences and differences in vascular physiology contribute to this difference [41, 42]. Despite these differences, a rise in mortality during the COVID‐19 period was observed in both sexes [21].

Differences observed across racial and ethnic groups highlight structural inequities and differences in preventive healthcare strategies [43, 44]. In this analysis, the racial and ethnic distribution of AAMRs, particularly the rate observed among NH White individuals, warrants cautious interpretation [45], since the substantial literature documenting a disproportionate cardiovascular burden among Black Americans would predict a different ranking. Several factors may contribute to this pattern. First, the proportion of deaths certified out of hospital at home, in hospice, or in long‐term care is higher among NH White and older decedents, settings in which ischemic and arrhythmic causes are frequently recorded without diagnostic confirmation and may be over‐attributed. Second, death‐certification and ICD‐10 coding practices may differ systematically across racial groups: greater prior contact with cardiology care among NH White decedents may increase the likelihood that an ischemic or arrhythmic diagnosis is recorded, whereas competing causes such as hypertensive, renal, or metabolic disease may be preferentially assigned as the underlying cause in other groups. Third, the NH White population has a substantially older age structure; although rates were standardized to the 2000 US population, age‐adjustment cannot fully remove residual differences when the cause is strongly age‐dependent, and a composite endpoint weighted toward conditions of advanced age may further accentuate the NH White estimate. Importantly, this pattern is not unique to the present analysis: Saad et al. reported the highest AAMR among NH White individuals for co‐occurring coronary artery disease and atrial fibrillation [24], and other CDC WONDER analyses of arrhythmia‐related mortality have observed the same [27]. This concordance suggests the finding reflects a reproducible feature of how ischemic and arrhythmic mortality is ascertained in these data rather than an anomaly of the present study. These differences may relate to socioeconomic factors, the prevalence of cardiovascular risk factors, healthcare accessibility, and comorbidities across racial and ethnic groups, although these factors were not directly measured in this study [46]. The influence of these existing structural inequities on cardiovascular outcomes across racial and ethnic populations has been extensively discussed in previously reported nationwide studies [47].

Elevated mortality was observed in the Southern and Midwest regions of the United States [48]. These characteristic patterns consistently align with prior studies, which exhibit the rising prevalence of cardiovascular risk factors, such as obesity, diabetes, and hypertension, in these prominent regions [49]. Most importantly, differences in socioeconomic status and healthcare infrastructure may also be associated with the regional variation observed [50], although these were not assessed directly. Our state‐level findings are consistent with those of Essa et al., who documented substantial variation in IHD age‐adjusted mortality and its trajectory across US states [19].

This manuscript highlights the influence of urbanization status on cardiovascular mortality patterns [51]. An elevated mortality trend was observed in non‐metropolitan areas [17, 51]. These differences may be attributable to limited access to specialized cardiac care, delays in emergency services, and decreased availability of advanced cardiac interventions within rural populations, although these factors were not directly investigated [18].

Mortality was concentrated among individuals aged ≥ 65 years [5]. These findings are consistent with previous studies that reflect the association of CVDs with aging [52]. Associative factors such as cardiometabolic risk factors accumulation, the rising prevalence of comorbidities like IHD and cardiac arrhythmias, and age‐related vascular changes in the older population of the United States [53]. Mortality among the youngest age group was low, despite the presence of cardiovascular risk factors in early adulthood [54].

The distribution of the places of death occurrence emphasizes highly insightful healthcare utilization patterns. The majority of deaths were accounted for within the medical facilities providing inpatient care, followed by deaths occurring at home or long‐term care facilities [14]. This prominent distribution aligns with prior studies and reflects the rising reliance on the management of hospital‐based acute cardiac events and the severity of cardiovascular conditions [55]. However, a considerable proportion of deaths occurring at home indicates the presentation of sudden cardiac events outside the clinical settings and delayed exposure to healthcare facilities [56].

## 5 Future Directions

Overall, substantial progress has been made over the past two decades in reducing cardiovascular mortality nationwide. Because of the observational design, causality cannot be inferred; the temporary surge in mortality observed during the COVID‐19 pandemic period is consistent with prior reports of major healthcare disruption [22]. Future studies should actively focus on targeted public health interventions, equitable healthcare access, and earlier cardiovascular risk assessment strategies associated with IHD and cardiac arrhythmias [57].

## 6 Limitations

Several limitations have been acknowledged with this manuscript. First, reliance of this analysis on death certificates from the CDC WONDER database may be subject to misclassification or inaccurate ICD‐10 coding for the underlying cause of death [58]. Second, the combination of two highly prevalent and epidemiologically distinct diseases limits the ability of the study to determine condition‐specific trends. Because both underlying and contributing causes of death were included, the absolute mortality counts reflect co‐occurring ischemic and arrhythmic mortality and are expected to exceed estimates based on underlying‐cause‐only definitions; they should therefore be interpreted as a measure of combined disease burden rather than cause‐specific mortality, and absolute rates should not be compared directly with underlying‐cause‐only statistics. Furthermore, cardiac arrhythmias are frequently recorded as a terminal mechanism rather than an underlying cause of death; while our requirement for concurrent IHD mitigates this issue, it does not eliminate it, and the mortality estimates should accordingly be interpreted with caution. Third, an observational study design limits the establishment of a causal relationship between the contributing factors and the existing mortality trend [59]. Fourth, the absence of patient‐level details such as disease severity, socioeconomic status, and existing comorbidities in the literature. Additionally, the upward mortality trend observed during the COVID‐19 pandemic period partially emphasizes the indirect effects of major healthcare disruptions [60]. Fifth, urban–rural analyses were limited to 2020 due to changes in the National Center for Health Statistics classification system, which redefined metropolitan categories and reclassified a proportion of US counties at the end of 2023 [61]. Lastly, formal statistical comparisons between subgroups were not performed; therefore, observed differences should be interpreted descriptively. Despite these existing limitations of this manuscript, the CDC WONDER database empowers the formation of a comprehensive assessment of long‐term mortality trends across the different subgroups of the US population.

## Author Contributions

M.M., S.M.E.A., and M.M.R. conceived and designed the study. M.F., L.I., A.A.R., and H.A. were responsible for data collection and literature search. S.K., Z.K., and S.T. performed the statistical analysis and interpreted the data. M.M. and A.S. drafted the initial manuscript. S.M.E.A., M.M.R., and A.S. critically revised the manuscript for important intellectual content. All authors (M.M., S.M.E.A., M.M.R., M.F., L.I., A.A.R., H.A., S.K., Z.K., S.T., A.S.) provided final approval of the version to be published and agreed to be accountable for all aspects of the work.

## Funding

The authors have nothing to report.

## Ethics Statement

The authors have nothing to report.

## Consent

The authors have nothing to report.

## Conflicts of Interest

The authors declare no conflicts of interest.

## Supporting information


Supporting File 1

## Data Availability

The data that support the findings of this study are openly available in CDC wonder at https://wonder.cdc.gov/. The data supporting this article are available in the article and its supplementary file. The datasets are publicly available for this study and can be accessed on the CDC WONDER website (https://wonder.cdc.gov/).
