# Supplementary material for: National Trends & Disparities in Ischemic Heart Disease & Cardiac Arrhythmias Related Mortality in the United States From 1999 to 2024: A CDC Wonder Analysis
Source: Clin Cardiol. 2026 Jul 27;49(8):e70427. doi: 10.1002/clc.70427 (PMC13405179; doi:10.1002/clc.70427)
Supplement: Supplementary file 1 — Supporting File 1 [file CLC-49-e70427-s001.docx]

***Supplemental File***

**Contents**

**Supplemental Table 1** Number of Ischemic heart disease and Cardiac Arrhythmia related Deaths, Stratified by Sex and Race in the United States, 1999-2024

**Supplemental Table 2** Number of Ischemic heart disease and Cardiac Arrhythmia related Deaths, Stratified by Urbanization and Census Region in the United States, 1999-2024

**Supplemental Table 3** Ischemic heart disease and Cardiac Arrhythmia related Age Adjusted Mortality Rates per 100,000 Stratified by Overall and Sex in the United States, 1999-2024

**Supplemental Table 4** Annual Percent Changes and Average Annual Percent Changes of Ischemic heart disease and Cardiac Arrhythmia related Mortality in United States from 1999 to 2024

**Supplemental Table 5** Ischemic heart disease and Cardiac Arrhythmia related Age Adjusted Mortality Rates per 100,000 Stratified by Race in the United States, 1999 to 2024

**Supplemental Table 6** Ischemic heart disease and Cardiac Arrhythmia related Age Adjusted Mortality Rates per 100,000 Stratified by Census Region in the United States, 1999 to 2024

**Supplemental Table 7** Ischemic heart disease and Cardiac Arrhythmia related Age Adjusted Mortality Rates per 100,000 Stratified by Urbanization in the United States, 1999 to 2020

**Supplemental Table 8** Ischemic heart disease and Cardiac Arrhythmia related Age Adjusted Mortality Rate per 100,000 stratified by States in the United States, 1999-2024

**Supplemental Table 9** Number of Ischemic heart disease and Cardiac Arrhythmia related Deaths, Place of Death in the United States, 1999-2024

**Supplemental Table 1** Number of Ischemic heart disease and Cardiac Arrhythmia related Deaths, Stratified by Sex and Race in the United States, 1999-2024

| **Deaths** | | | | | | | | |
| --- | --- | --- | --- | --- | --- | --- | --- | --- |
| **Year** | **Overall** | **Female** | **Male** | **NH White** | **NH Black or African American** | **NH Asian or Pacific Islander** | **NH American Indian or Alaska Native** | **Hispanic or Latino** |
| 1999 | 26919 | 14332 | 12587 | 23257 | 2293 | 313 | 101 | 891 |
| 2000 | 26996 | 14341 | 12655 | 23270 | 2355 | 299 | 86 | 927 |
| 2001 | 25900 | 13839 | 12061 | 22284 | 2212 | 297 | 67 | 980 |
| 2002 | 25314 | 13418 | 11896 | 21519 | 2253 | 382 | 104 | 989 |
| 2003 | 24501 | 12903 | 11598 | 20995 | 2047 | 311 | 118 | 983 |
| 2004 | 23270 | 12202 | 11068 | 19833 | 2010 | 328 | 106 | 953 |
| 2005 | 23002 | 12076 | 10926 | 19387 | 2149 | 381 | 64 | 986 |
| 2006 | 21506 | 11125 | 10381 | 18042 | 1944 | 393 | 93 | 993 |
| 2007 | 20445 | 10638 | 9807 | 17074 | 1907 | 378 | 85 | 982 |
| 2008 | 20116 | 10411 | 9705 | 16860 | 1827 | 340 | 81 | 974 |
| 2009 | 18901 | 9574 | 9327 | 15775 | 1711 | 367 | 100 | 922 |
| 2010 | 18354 | 9271 | 9083 | 15277 | 1709 | 372 | 74 | 894 |
| 2011 | 18336 | 9026 | 9310 | 15192 | 1714 | 352 | 105 | 952 |
| 2012 | 18238 | 8965 | 9273 | 15066 | 1696 | 378 | 106 | 959 |
| 2013 | 18126 | 8746 | 9380 | 14815 | 1779 | 413 | 98 | 978 |
| 2014 | 18466 | 8745 | 9721 | 14951 | 1835 | 431 | 114 | 1090 |
| 2015 | 18941 | 9033 | 9908 | 15197 | 1943 | 476 | 101 | 1165 |
| 2016 | 19256 | 8857 | 10399 | 15389 | 1982 | 470 | 128 | 1232 |
| 2017 | 19996 | 9166 | 10830 | 15803 | 2104 | 560 | 120 | 1355 |
| 2018 | 20362 | 9018 | 11344 | 16101 | 2080 | 579 | 137 | 1414 |
| 2019 | 20611 | 9044 | 11567 | 16072 | 2276 | 531 | 159 | 1542 |
| 2020 | 22628 | 9776 | 12852 | 17258 | 2624 | 656 | 189 | 1862 |
| 2021 | 24102 | 10395 | 13707 | 18407 | 2755 | 700 | 180 | 1922 |
| 2022 | 23688 | 10211 | 13477 | 18181 | 2688 | 697 | 157 | 1838 |
| 2023 | 22508 | 9540 | 12968 | 17087 | 2734 | 622 | 134 | 1785 |
| 2024 | 22375 | 9498 | 12877 | 16787 | 2757 | 713 | 178 | 1786 |
| NH: Non-Hispanic | | | | | | | | |

**Supplemental Table 2** Number of Ischemic heart disease and Cardiac Arrhythmia related Deaths, Stratified by Urbanization and Census Region in the United States, 1999-2024

|  | | | | | | |
| --- | --- | --- | --- | --- | --- | --- |
| **Deaths** | | | | | | |
| **Year** | **Metropolitan** | **Non-Metropolitan** | **Northeast** | **Midwest** | **South** | **West** |
| 1999 | 58641 | 15203 | 15818 | 19311 | 25000 | 13715 |
| 2000 | 53936 | 14329 | 14823 | 17853 | 22641 | 12948 |
| 2001 | 56471 | 14409 | 15608 | 17844 | 23587 | 13841 |
| 2002 | 56099 | 14373 | 15232 | 17816 | 23401 | 14023 |
| 2003 | 54156 | 14243 | 14636 | 17110 | 22996 | 13657 |
| 2004 | 50960 | 13143 | 13703 | 16367 | 21178 | 12855 |
| 2005 | 52262 | 13551 | 13631 | 16891 | 22113 | 13178 |
| 2006 | 50198 | 13006 | 12615 | 16425 | 21113 | 13051 |
| 2007 | 49233 | 12896 | 12759 | 16134 | 20635 | 12601 |
| 2008 | 50141 | 12979 | 12889 | 16375 | 20662 | 13194 |
| 2009 | 48560 | 12748 | 12272 | 15792 | 20530 | 12714 |
| 2010 | 49642 | 12942 | 12895 | 15588 | 20856 | 13245 |
| 2011 | 51350 | 12853 | 13115 | 16132 | 21044 | 13912 |
| 2012 | 51723 | 13345 | 13116 | 16033 | 21936 | 13983 |
| 2013 | 53476 | 13360 | 13429 | 16412 | 22575 | 14420 |
| 2014 | 53951 | 13945 | 13385 | 16572 | 23362 | 14577 |
| 2015 | 56577 | 14348 | 13567 | 17227 | 24533 | 15598 |
| 2016 | 56447 | 14171 | 13125 | 17020 | 24487 | 15986 |
| 2017 | 59375 | 14959 | 13473 | 18128 | 26091 | 16642 |
| 2018 | 61386 | 15885 | 13853 | 18741 | 27621 | 17056 |
| 2019 | 62406 | 16461 | 13946 | 18841 | 28774 | 17306 |
| 2020 | 71588 | 18455 | 16187 | 21561 | 33316 | 18979 |
| 2021 | - | - | 15922 | 22189 | 35121 | 19930 |
| 2022 | - | - | 15906 | 21500 | 35403 | 20325 |
| 2023 | - | - | 15108 | 20382 | 34809 | 19141 |
| 2024 | - | - | 14770 | 20917 | 35279 | 19033 |
|  | | | | | | |

**Supplemental Table 3** Ischemic heart disease and Cardiac Arrhythmia related Age Adjusted Mortality Rates per 100,000 Stratified by Overall and Sex in the United States, 1999-2024

|  | | | |
| --- | --- | --- | --- |
| **Age Adjusted Mortality Rates per 100,000 (95% CI)** | | | |
| **Year** | **Overall** | **Female** | **Male** |
| 1999 | 41.97 (41.66 – 42.27) | 31.72 (31.39 – 32.05) | 56.50 (55.92 – 57.07) |
| 2000 | 38.29 (38.01 – 38.58) | 28.99 (28.67 – 29.30) | 51.54 (50.99 – 52.09) |
| 2001 | 39.10 (38.81 – 39.38) | 29.65 (29.34 – 29.97) | 52.55 (52.01 – 53.10) |
| 2002 | 38.30 (38.01 – 38.58) | 29.16 (28.84 – 29.47) | 51.29 (50.75 – 51.82) |
| 2003 | 36.55 (36.27 – 36.82) | 27.81 (27.50 – 28.12) | 48.98 (48.46 – 49.49) |
| 2004 | 33.75 (33.49 – 34.01) | 25.52 (25.23 – 25.81) | 45.52 (45.02 – 46.01) |
| 2005 | 33.97 (33.71 – 34.23) | 25.81 (25.52 – 26.10) | 45.65 (45.16 – 46.14) |
| 2006 | 31.95 (31.70 – 32.20) | 24.10 (23.83 – 24.38) | 43.00 (42.53 – 43.47) |
| 2007 | 30.73 (30.49 – 30.97) | 23.04 (22.77 – 23.30) | 41.63 (41.17 – 42.08) |
| 2008 | 30.58 (30.34 – 30.81) | 23.05 (22.78 – 23.32) | 41.21 (40.76 – 41.65) |
| 2009 | 29.13 (28.90 – 29.36) | 21.53 (21.27 – 21.78) | 39.75 (39.32 – 40.18) |
| 2010 | 29.27 (29.04 – 29.50) | 21.31 (21.06 – 21.57) | 40.39 (39.96 – 40.82) |
| 2011 | 29.15 (28.92 – 29.37) | 21.31 (21.06 – 21.56) | 40.05 (39.63 – 40.48) |
| 2012 | 28.81 (28.59 – 29.04) | 20.91 (20.66 – 21.15) | 39.88 (39.46 – 40.29) |
| 2013 | 28.86 (28.64 – 29.08) | 20.69 (20.45 – 20.94) | 40.28 (39.87 – 40.70) |
| 2014 | 28.70 (28.49 – 28.92) | 20.23 (19.99 – 20.46) | 40.24 (39.83 – 40.64) |
| 2015 | 29.30 (29.09 – 29.52) | 20.61 (20.37 – 20.85) | 41.16 (40.76 – 41.57) |
| 2016 | 28.55 (28.34 – 28.77) | 19.81 (19.58 – 20.04) | 40.54 (40.14 – 40.93) |
| 2017 | 29.37 (29.16 – 29.59) | 20.04 (19.81 – 20.27) | 41.99 (41.59 – 42.39) |
| 2018 | 29.84 (29.63 – 30.05) | 20.31 (20.08 – 20.53) | 42.77 (42.37 – 43.16) |
| 2019 | 29.81 (29.60 – 30.02) | 20.11 (19.89 – 20.34) | 42.85 (42.46 – 43.24) |
| 2020 | 33.42 (33.20 – 33.64) | 22.27 (22.04 – 22.51) | 48.48 (48.07 – 48.89) |
| 2021 | 36.06 (35.83 – 36.30) | 23.95 (23.70 – 24.20) | 52.21 (51.78 – 52.65) |
| 2022 | 34.04 (33.82 – 34.26) | 22.33 (22.10 – 22.57) | 50.14 (49.73 – 50.56) |
| 2023 | 32.73 (32.51 – 32.95) | 21.57 (21.34 – 21.81) | 47.62 (47.21 – 48.02) |
| 2024 | 31.75 (31.54 – 31.96) | 20.77 (20.55 – 20.99) | 46.21 (45.82 – 46.60) |
| **Total** | **32.46 (32.22 – 32.70)** | **23.33 (23.07 – 23.59)** | **45.09 (44.65 – 45.54)** |

CI: Confidence Interval

**Supplemental Table 4** Annual Percent Changes and Average Annual Percent Changes of Ischemic heart disease and Cardiac Arrhythmia related Mortality in United States from 1999 to 2024

| **Year Interval** | **APC (95% CI); p-value** | **AAPC (95% CI); p-value** |
| --- | --- | --- |
| **Overall** | | |
| 1999-2009 | -3.49 (-4.00 to -3.09); < 0.000001 | -1.08 (-1.24 to -0.95); < 0.000001 |
| 2009-2018 | -0.03 (-0.69 to 0.57); 0.870226 |  |
| 2018-2021 | 7.08 (4.69 to 8.32); < 0.000001 |  |
| 2021-2024 | -3.90 (-5.49 to -2.52); < 0.000001 |  |
| **Male** | | |
| 1999-2009 | -3.35 (-3.88 to -2.94); < 0.000001 | -0.74 (-0.90 to -0.60); < 0.000001 |
| 2009-2018 | 0.59 (-0.06 to 1.15); 0.067586 |  |
| 2018-2021 | 7.61 (5.48 to 8.82); < 0.000001 |  |
| 2021-2024 | -3.85 (-5.32 to -2.50); < 0.000001 |  |
| **Female** | | |
| 1999-2009 | -3.69 (-4.42 to -3.24); < 0.000001 | -1.68 (-1.89 to -1.53); < 0.000001 |
| 2009-2018 | -1.09 (-1.86 to -0.30); 0.012797 |  |
| 2018-2021 | 6.21 (3.36 to 7.66); < 0.000001 |  |
| 2021-2024 | -4.27 (-6.73 to -2.62); < 0.000001 |  |
| **NH Black or African American** | | |
| 1999-2010 | -4.22 (-5.10 to -3.71); 0.0012 | -1.68 (-1.93 to -1.49); < 0.000001 |
| 2010-2018 | -0.57 (-2.23 to 0.57); 0.234753 |  |
| 2018-2021 | 6.19 (2.91 to 8.04); 0.005599 |  |
| 2021-2024 | -2.72 (-6.50 to -0.67); 0.019196 |  |
| **NH Asian or Pacific Islander** | | |
| 1999-2015 | -2.76 (-3.53 to -2.11); 0.007199 | -1.65 (-2.03 to -1.29); < 0.000001 |
| 2015-2021 | 3.19 (1.35 to 7.94); 0.016797 |  |
| 2021-2024 | -5.04 (-10.65 to -1.59); 0.015997 |  |
| **NH American Indian or Alaska Native** | | |
| 1999-2024 | -0.39 (-0.91 to 0.22); 0.228754 | -0.3934 (-0.91 to 0.22); 0.228754 |
| **NH Whites** | | |
| 1999-2009 | -3.24 (-3.75 to -2.83); < 0.000001 | -0.72 (-0.89 to -0.59); < 0.000001 |
| 2009-2018 | 0.33 (-0.31 to 0.93); 0.278344 |  |
| 2018-2021 | 7.56 (5.07 to 8.84); < 0.000001 |  |
| 2021-2024 | -3.29 (-4.98 to -1.86); < 0.000001 |  |
| **Hispanic or Latino** | | |
| 1999-2014 | -3.76 (-4.59 to -3.03); 0.0008 | -1.92 (-2.34 to -1.53); < 0.000001 |
| 2014-2021 | 4.17 (2.53 to 8.94); 0.002799 |  |
| 2021-2024 | -6.30 (-12.41 to -2.17); 0.003199 |  |
| **Northeast** | | |
| 1999-2007 | -4.18 (-5.21 to -3.45); < 0.000001 | -1.70 (-1.93 to -1.51); < 0.000001 |
| 2007-2018 | -0.92 (-1.57 to -0.31); 0.012398 |  |
| 2018-2021 | 5.89 (2.69 to 7.47); < 0.000001 |  |
| 2021-2024 | -5.13 (-8.21 to -3.22); < 0.000001 |  |
| **Midwest** | | |
| 1999-2010 | -3.02 (-4.12 to -2.51); 0.004399 | -0.97 (-1.25 to -0.78); < 0.000001 |
| 2010-2018 | 0.30 (-1.48 to 1.57); 0.635073 |  |
| 2018-2021 | 6.90 (3.52 to 8.64); < 0.000001 |  |
| 2021-2024 | -4.24 (-7.40 to -2.42); 0.0008 |  |
| **South** | | |
| 1999-2009 | -3.82 (-4.47 to -3.33); < 0.000001 | -0.79 (-0.98 to -0.63); < 0.000001 |
| 2009-2018 | 0.28 (-0.51 to 0.97); 0.413917 |  |
| 2018-2021 | 8.87 (6.03 to 10.33); < 0.000001 |  |
| 2021-2024 | -2.91 (-4.90 to -1.33); 0.002 |  |
| **West** | | |
| 1999-2009 | -3.15 (-4.33 to -2.57); 0.002799 | -1.15 (-1.40 to -0.95); < 0.000001 |
| 2009-2018 | 0.03 (-1.43 to 0.94); 0.90182 |  |
| 2018-2021 | 5.44 (2.38 to 7.02); < 0.000001 |  |
| 2021-2024 | -4.22 (-7.29 to -2.31); < 0.000001 |  |
| **Metropolitan** | | |
| 1999-2009 | -3.64 (-4.28 to -3.17); 0.0004 | -1.25 (-1.51 to -1.07); < 0.000001 |
| 2009-2018 | -0.13 (-1.20 to 0.53); 0.559888 |  |
| 2018-2020 | 6.09 (2.05 to 8.25); < 0.000001 |  |
| **Non-Metropolitan** | | |
| 1999-2009 | -2.82 (-3.56 to -2.35); 0.0004 | -0.41 (-0.65 to -0.22); < 0.000001 |
| 2009-2017 | 0.41 (-0.84 to 1.37); 0.359928 |  |
| 2017-2020 | 5.70 (3.28 to 9.08); < 0.000001 |  |
| **Alabama** | | |
| 1999–2009 | -3.40 (-6.74 to -2.04); <0.000001 | -1.21 (-1.63 to -0.77); <0.000001 |
| 2009–2024 | 0.28 (-0.46 to 1.79); 0.371 |  |
| **Alaska** | | |
| 1999–2002 | -18.46 (-32.73 to -4.15); 0.002 | -2.10 (-2.91 to -0.61); 0.010 |
| 2002–2024 | 0.37 (-0.45 to 1.93); 0.249 |  |
| **Arizona** | | |
| 1999–2007 | -3.93 (-9.06 to -1.13); 0.005 | 0.83 (0.20 to 1.47); 0.011 |
| 2007–2024 | 3.15 (2.37 to 4.23); <0.000001 |  |
| **Arkansas** | | |
| 1999–2013 | -2.36 (-3.62 to -1.02); 0.033 | 0.54 (0.08 to 1.16); 0.032 |
| 2013–2022 | 6.13 (-3.92 to 11.48); 0.126 |  |
| 2022–2024 | -3.31 (-8.89 to 5.22); 0.559 |  |
| **California** | | |
| 1999–2010 | -3.39 (-5.31 to -2.51); <0.000001 | -2.03 (-2.35 to -1.70); <0.000001 |
| 2010–2024 | -0.95 (-1.55 to 0.28); 0.079 |  |
| **Colorado** | | |
| 1999–2012 | -3.52 (-4.41 to -2.80); <0.000001 | 0.46 (0.12 to 0.80); 0.022 |
| 2012–2022 | 8.15 (7.43 to 9.43); <0.000001 |  |
| 2022–2024 | -9.63 (-13.93 to -3.83); <0.001 |  |
| **Connecticut** | | |
| 1999–2001 | 3.01 (-5.14 to 9.00); 0.511 | -2.68 (-3.10 to -2.27); <0.000001 |
| 2001–2008 | -6.90 (-10.96 to -2.23); 0.026 |  |
| 2008–2024 | -1.48 (-2.17 to -0.75); 0.013 |  |
| **Delaware** | | |
| 1999–2011 | -4.44 (-10.00 to -2.61); 0.001 | -1.90 (-2.67 to -1.12); <0.000001 |
| 2011–2024 | 0.51 (-1.07 to 5.49); 0.443 |  |
| **District of Columbia** | | |
| 1999–2014 | -4.06 (-6.85 to -2.69); <0.001 | -1.47 (-2.34 to -0.64); 0.001 |
| 2014–2024 | 2.55 (-0.20 to 9.67); 0.070 |  |
| **Florida** | | |
| 1999–2011 | -4.39 (-5.40 to -3.78); 0.002 | -0.99 (-1.29 to -0.74); <0.000001 |
| 2011–2017 | 1.40 (-2.40 to 3.77); 0.296 |  |
| 2017–2021 | 8.73 (5.73 to 12.17); <0.000001 |  |
| 2021–2024 | -4.15 (-7.65 to -1.01); 0.004 |  |
| **Georgia** | | |
| 1999–2007 | -6.65 (-11.48 to -4.60); 0.019 | -2.16 (-2.60 to -1.70); <0.000001 |
| 2007–2016 | -1.72 (-6.17 to 0.52); 0.095 |  |
| 2016–2024 | 2.03 (0.19 to 7.59); 0.038 |  |
| **Hawaii** | | |
| 1999–2008 | -0.76 (-3.09 to 7.56); 0.669 | -1.39 (-2.26 to -0.48); 0.013 |
| 2008–2012 | -7.55 (-13.12 to 4.10); 0.096 |  |
| 2012–2024 | 0.28 (-5.91 to 6.34); 0.527 |  |
| **Idaho** | | |
| 1999–2009 | -3.75 (-11.16 to -1.20); 0.002 | -0.47 (-1.25 to 0.41); 0.252 |
| 2009–2024 | 1.78 (0.61 to 5.20); 0.004 |  |
| **Illinois** | | |
| 1999–2012 | -3.56 (-6.47 to 0.84); 0.056 | -2.47 (-2.97 to -2.10); <0.000001 |
| 2012–2018 | -1.05 (-6.59 to 0.95); 0.159 |  |
| 2018–2021 | 6.47 (1.41 to 9.48); 0.002 |  |
| 2021–2024 | -8.89 (-14.00 to -5.82); <0.000001 |  |
| **Indiana** | | |
| 1999–2009 | -3.67 (-5.57 to -2.46); 0.008 | -0.12 (-0.58 to 0.23); 0.448 |
| 2009–2012 | 9.93 (2.65 to 13.36); 0.014 |  |
| 2012–2024 | 0.50 (-1.45 to 1.33); 0.510 |  |
| **Iowa** | | |
| 1999–2012 | -2.30 (-3.08 to -1.63); <0.000001 | -0.22 (-0.63 to 0.11); 0.192 |
| 2012–2021 | 4.47 (3.41 to 6.91); 0.001 |  |
| 2021–2024 | -4.74 (-11.49 to -0.72); 0.022 |  |
| **Kansas** | | |
| 1999–2012 | -4.40 (-5.57 to -3.42); <0.001 | -0.79 (-1.37 to -0.33); 0.003 |
| 2012–2020 | 6.95 (4.98 to 12.97); 0.005 |  |
| 2020–2024 | -3.67 (-10.56 to 0.42); 0.076 |  |
| **Kentucky** | | |
| 1999–2009 | -2.47 (-4.32 to -1.37); 0.001 | -0.64 (-1.13 to -0.25); 0.005 |
| 2009–2022 | 2.29 (1.70 to 3.71); 0.001 |  |
| 2022–2024 | -9.80 (-15.51 to -3.14); 0.005 |  |
| **Louisiana** | | |
| 1999–2007 | -5.83 (-12.43 to -2.89); <0.001 | 0.84 (0.21 to 1.57); 0.011 |
| 2007–2024 | 4.14 (3.14 to 5.43); <0.000001 |  |
| **Maine** | | |
| 1999–2005 | -4.67 (-9.45 to -2.88); <0.000001 | -0.55 (-0.96 to -0.20); 0.007 |
| 2005–2017 | -1.07 (-1.89 to 0.59); 0.105 |  |
| 2017–2021 | 11.07 (7.74 to 15.89); <0.000001 |  |
| 2021–2024 | -4.59 (-10.03 to -0.91); 0.012 |  |
| **Maryland** | | |
| 1999–2013 | -4.74 (-7.09 to -1.17); 0.040 | -1.31 (-1.75 to -0.94); <0.000001 |
| 2013–2017 | 1.26 (-6.02 to 5.63); 0.822 |  |
| 2017–2021 | 10.62 (6.69 to 15.76); 0.001 |  |
| 2021–2024 | -3.39 (-8.29 to 0.33); 0.070 |  |
| **Massachusetts** | | |
| 1999–2013 | -3.68 (-4.52 to -3.00); 0.013 | -1.25 (-1.67 to -0.87); <0.000001 |
| 2013–2021 | 3.64 (-3.54 to 8.27); 0.094 |  |
| 2021–2024 | -2.47 (-8.77 to 2.31); 0.282 |  |
| **Michigan** | | |
| 1999–2006 | -6.14 (-7.56 to -5.12); <0.000001 | -2.05 (-2.35 to -1.81); <0.000001 |
| 2006–2017 | -0.97 (-1.77 to -0.27); 0.012 |  |
| 2017–2021 | 5.90 (3.19 to 9.11); <0.000001 |  |
| 2021–2024 | -6.37 (-9.69 to -3.26); <0.000001 |  |
| **Minnesota** | | |
| 1999–2002 | -0.79 (-4.62 to 5.10); 0.659 | 0.82 (0.51 to 1.25); <0.000001 |
| 2002–2006 | -6.83 (-10.28 to 3.27); 0.120 |  |
| 2006–2015 | 0.84 (-1.10 to 4.66); 0.168 |  |
| 2015–2021 | 8.53 (6.97 to 12.20); <0.000001 |  |
| 2021–2024 | -1.81 (-5.14 to 0.80); 0.152 |  |
| **Mississippi** | | |
| 1999–2016 | -1.58 (-2.34 to -0.87); <0.001 | 1.51 (1.02 to 1.97); <0.000001 |
| 2016–2021 | 15.53 (11.45 to 23.73); <0.001 |  |
| 2021–2024 | -2.54 (-10.12 to 2.85); 0.343 |  |
| **Missouri** | | |
| 1999–2004 | -0.77 (-3.15 to 5.02); 0.643 | -1.60 (-1.98 to -1.21); <0.000001 |
| 2004–2016 | -4.58 (-7.95 to -3.80); 0.024 |  |
| 2016–2024 | 2.50 (0.93 to 4.48); 0.019 |  |
| **Montana** | | |
| 1999–2009 | -3.97 (-14.88 to 14.29); 0.097 | -0.41 (-1.73 to 1.06); 0.259 |
| 2009–2018 | 1.60 (-12.02 to 7.01); 0.583 |  |
| 2018–2021 | 16.32 (4.85 to 24.66); 0.020 |  |
| 2021–2024 | -9.36 (-23.13 to -1.15); 0.041 |  |
| **Nebraska** | | |
| 1999–2014 | 0.75 (-5.70 to 2.00); 0.530 | 2.01 (1.27 to 2.83); <0.000001 |
| 2014–2024 | 3.94 (2.16 to 11.51); 0.018 |  |
| **Nevada** | | |
| 1999–2013 | -3.98 (-5.53 to -2.73); 0.002 | 1.15 (0.63 to 1.71); <0.000001 |
| 2013–2017 | 16.53 (9.35 to 25.55); 0.007 |  |
| 2017–2024 | 3.54 (0.29 to 5.48); 0.041 |  |
| **New Hampshire** | | |
| 1999–2008 | -5.62 (-9.62 to -3.79); <0.000001 | -2.10 (-2.61 to -1.56); <0.000001 |
| 2008–2024 | -0.07 (-0.91 to 1.21); 0.968 |  |
| **New Jersey** | | |
| 1999–2008 | -5.79 (-6.94 to -4.95); 0.002 | -2.62 (-2.98 to -2.34); <0.000001 |
| 2008–2011 | 7.36 (2.25 to 9.46); 0.027 |  |
| 2011–2021 | -1.24 (-2.00 to -0.22); 0.037 |  |
| 2021–2024 | -6.90 (-12.34 to -3.69); <0.000001 |  |
| **New Mexico** | | |
| 1999–2013 | -1.56 (-2.93 to -0.53); 0.004 | 0.26 (-0.29 to 0.75); 0.263 |
| 2013–2020 | 6.98 (4.77 to 14.86); <0.000001 |  |
| 2020–2024 | -4.61 (-10.86 to -0.82); 0.016 |  |
| **New York** | | |
| 1999–2017 | -1.97 (-2.49 to -1.50); 0.009 | -1.01 (-1.45 to -0.66); 0.004 |
| 2017–2021 | 6.87 (3.11 to 11.54); 0.024 |  |
| 2021–2024 | -5.23 (-11.80 to -1.18); 0.027 |  |
| **North Carolina** | | |
| 1999–2004 | -5.01 (-9.62 to -2.74); 0.019 | -0.83 (-1.24 to -0.40); 0.003 |
| 2004–2019 | -2.12 (-3.09 to 1.21); 0.100 |  |
| 2019–2022 | 12.20 (-5.61 to 14.67); 0.244 |  |
| 2022–2024 | 1.24 (-4.50 to 8.87); 0.481 |  |
| **North Dakota** | | |
| 1999–2008 | 0.38 (-0.71 to 3.73); 0.469 | -1.40 (-1.89 to -0.90); <0.000001 |
| 2008–2014 | -4.49 (-9.97 to -2.26); 0.002 |  |
| 2014–2020 | 4.14 (1.78 to 10.82); 0.002 |  |
| 2020–2024 | -8.47 (-14.51 to -4.80); <0.001 |  |
| **Ohio** | | |
| 1999–2018 | -2.03 (-2.37 to -1.73); 0.016 | -1.26 (-1.58 to -1.02); <0.000001 |
| 2018–2021 | 5.73 (-2.17 to 7.61); 0.074 |  |
| 2021–2024 | -3.09 (-8.19 to 0.96); 0.078 |  |
| **Oklahoma** | | |
| 1999–2002 | 4.90 (-1.46 to 16.27); 0.140 | 1.38 (0.87 to 2.04); <0.000001 |
| 2002–2011 | -2.95 (-9.47 to 5.89); 0.088 |  |
| 2011–2014 | 8.35 (-2.21 to 12.45); 0.120 |  |
| 2014–2024 | 2.30 (-3.28 to 5.19); 0.093 |  |
| **Oregon** | | |
| 1999–2004 | -3.73 (-10.62 to -0.38); 0.031 | 1.30 (0.80 to 1.86); <0.000001 |
| 2004–2016 | 1.24 (-0.23 to 3.89); 0.072 |  |
| 2016–2022 | 7.90 (6.25 to 13.76); 0.009 |  |
| 2022–2024 | -4.39 (-10.79 to 1.74); 0.131 |  |
| **Pennsylvania** | | |
| 1999–2010 | -4.27 (-4.90 to -3.83); 0.002 | -1.73 (-1.97 to -1.56); <0.000001 |
| 2010–2013 | 3.35 (0.39 to 5.07); 0.032 |  |
| 2013–2018 | -2.70 (-5.79 to -1.57); <0.001 |  |
| 2018–2021 | 7.89 (4.19 to 10.10); <0.000001 |  |
| 2021–2024 | -4.71 (-7.40 to -2.86); <0.000001 |  |
| **Rhode Island** | | |
| 1999–2010 | -4.13 (-6.54 to -2.93); <0.000001 | -1.54 (-2.02 to -1.09); <0.000001 |
| 2010–2024 | 0.54 (-0.45 to 2.15); 0.236 |  |
| **South Carolina** | | |
| 1999–2010 | -4.47 (-5.49 to -3.59); <0.000001 | -0.62 (-0.88 to -0.34); <0.000001 |
| 2010–2024 | 2.51 (1.95 to 3.20); <0.000001 |  |
| **South Dakota** | | |
| 1999–2010 | -1.24 (-4.56 to 0.44); 0.143 | 1.64 (1.08 to 2.24); <0.000001 |
| 2010–2024 | 3.96 (2.93 to 5.83); <0.000001 |  |
| **Tennessee** | | |
| 1999–2008 | -0.73 (-1.27 to 0.42); 0.122 | -0.49 (-0.70 to -0.28); <0.001 |
| 2008–2017 | -2.59 (-4.42 to -2.07); <0.000001 |  |
| 2017–2021 | 6.99 (4.99 to 9.92); <0.000001 |  |
| 2021–2024 | -2.98 (-5.60 to -0.61); 0.013 |  |
| **Texas** | | |
| 1999–2003 | -1.30 (-3.04 to 2.43); 0.345 | -0.99 (-1.23 to -0.70); <0.000001 |
| 2003–2007 | -7.72 (-10.66 to -5.56); 0.008 |  |
| 2007–2018 | 1.10 (0.00 to 1.64); 0.051 |  |
| 2018–2021 | 5.62 (2.67 to 7.14); <0.000001 |  |
| 2021–2024 | -5.13 (-7.93 to -3.11); <0.000001 |  |
| **Utah** | | |
| 1999–2008 | -5.06 (-11.54 to -0.85); 0.044 | -0.80 (-1.63 to 0.19); 0.081 |
| 2008–2022 | 3.10 (-6.98 to 12.81); 0.121 |  |
| 2022–2024 | -7.78 (-17.44 to 2.68); 0.311 |  |
| **Vermont** | | |
| 1999–2004 | -5.32 (-14.92 to -0.85); 0.028 | -1.54 (-2.18 to -0.61); 0.002 |
| 2004–2024 | -0.57 (-5.05 to 4.31); 0.521 |  |
| **Virginia** | | |
| 1999–2007 | -4.66 (-10.08 to -3.02); 0.008 | -0.24 (-0.76 to 0.18); 0.218 |
| 2007–2018 | -0.83 (-2.22 to 2.03); 0.316 |  |
| 2018–2021 | 15.71 (9.46 to 19.23); 0.022 |  |
| 2021–2024 | -0.78 (-7.08 to 2.77); 0.589 |  |
| **Washington** | | |
| 1999–2006 | -1.98 (-6.34 to -0.48); 0.003 | -0.23 (-0.56 to 0.13); 0.174 |
| 2006–2024 | 0.46 (0.14 to 1.34); 0.010 |  |
| **West Virginia** | | |
| 1999–2014 | -2.54 (-4.18 to -1.62); <0.000001 | -0.50 (-1.04 to 0.03); 0.062 |
| 2014–2024 | 2.64 (0.92 to 7.13); 0.004 |  |
| **Wisconsin** | | |
| 1999–2011 | -3.80 (-4.76 to -2.96); 0.003 | 0.30 (-0.10 to 0.67); 0.124 |
| 2011–2021 | 5.96 (4.95 to 8.41); 0.019 |  |
| 2021–2024 | -1.28 (-7.61 to 3.04); 0.543 |  |
| **Wyoming** | | |
| 1999–2013 | -3.75 (-6.98 to -1.61); 0.008 | -0.38 (-1.70 to 0.60); 0.451 |
| 2013–2021 | 10.72 (6.98 to 29.31); 0.010 |  |
| 2021–2024 | -11.71 (-26.39 to -2.18); 0.023 |  |

APC: Annual Percent Change, AAPC: Average Annual Percent Change, CI: Confidence Interval, NH: Non-Hispanic

**Supplemental Table 5** Ischemic heart disease and Cardiac Arrhythmia related Age Adjusted Mortality Rates per 100,000 Stratified by Race in the United States, 1999 to 2024

|  | | | | | |
| --- | --- | --- | --- | --- | --- |
| **Age Adjusted Mortality Rates per 100,000 (95% CI)** | | | | | |
| **Year** | **American Indian or Alaska Native** | **Asian or Pacific Islander** | **Black or African American** | **Hispanic or Latino** | **White** |
| 1999 | 33.03 (28.49 – 37.57) | 21.13 (19.57 – 22.68) | 37.42 (36.41 – 38.42) | 28.48 (27.25 – 29.70) | 43.47 (43.14 – 43.81) |
| 2000 | 28.14 (24.20 – 32.09) | 17.61 (16.24 – 18.97) | 33.20 (32.26 – 34.14) | 24.86 (23.75 – 25.98) | 39.90 (39.58 – 40.22) |
| 2001 | 26.78 (22.99 – 30.56) | 21.71 (20.25 – 23.17) | 34.56 (33.60 – 35.51) | 29.03 (27.86 – 30.2) | 40.49 (40.17 – 40.81) |
| 2002 | 26.80 (22.98 – 30.62) | 21.90 (20.50 – 23.30) | 33.70 (32.76 – 34.63) | 26.85 (25.75 – 27.96) | 39.79 (39.48 – 40.11) |
| 2003 | 29.66 (25.68 – 33.65) | 20.18 (18.85 – 21.50) | 32.87 (31.95 – 33.79) | 25.64 (24.59 – 26.70) | 38.01 (37.71 – 38.32) |
| 2004 | 29.48 (25.55 – 33.40) | 18.14 (16.92 – 19.35) | 30.39 (29.51 – 31.27) | 24.35 (23.35 – 25.35) | 35.13 (34.84 – 35.42) |
| 2005 | 23.72 (20.25 – 27.19) | 17.73 (16.58 – 18.88) | 29.52 (28.66 – 30.37) | 24.58 (23.61 – 25.55) | 35.49 (35.20 – 35.78) |
| 2006 | 29.09 (25.30 – 32.87) | 17.62 (16.50 – 18.73) | 27.67 (26.85 – 28.49) | 21.98 (21.08 – 22.88) | 33.44 (33.16 – 33.72) |
| 2007 | 23.90 (20.52 – 27.28) | 14.83 (13.84 – 15.81) | 26.07 (25.29 – 26.86) | 19.28 (18.46 – 20.09) | 32.54 (32.26 – 32.81) |
| 2008 | 23.80 (20.48 – 27.11) | 16.86 (15.83 – 17.89) | 25.07 (24.31 – 25.82) | 20.09 (19.28 – 20.9) | 32.32 (32.05 – 32.59) |
| 2009 | 27.76 (24.24 – 31.27) | 15.42 (14.46 – 16.37) | 23.72 (22.99 – 24.45) | 18.72 (17.96 – 19.48) | 30.90 (30.63 – 31.16) |
| 2010 | 26.72 (23.32 – 30.12) | 15.88 (14.94 – 16.83) | 23.20 (22.49 – 23.92) | 19.12 (18.36 – 19.87) | 31.13 (30.87 – 31.39) |
| 2011 | 25.66 (22.43 – 28.9) | 15.29 (14.40 – 16.18) | 22.84 (22.15 – 23.54) | 17.76 (17.07 – 18.45) | 31.23 (30.97 – 31.49) |
| 2012 | 26.92 (23.73 – 30.12) | 14.97 (14.13 – 15.82) | 23.09 (22.40 – 23.78) | 17.22 (16.55 – 17.88) | 30.92 (30.66 – 31.18) |
| 2013 | 22.25 (19.46 – 25.04) | 14.87 (14.05 – 15.68) | 22.9 (22.23 – 23.57) | 17.69 (17.04 – 18.34) | 31.08 (30.82 – 31.34) |
| 2014 | 24.77 (21.88 – 27.67) | 13.80 (13.05 – 14.56) | 22.93 (22.27 – 23.59) | 17.41 (16.79 – 18.04) | 30.98 (30.72 – 31.23) |
| 2015 | 25.56 (22.71 – 28.4) | 13.94 (13.21 – 14.67) | 22.62 (21.98 – 23.27) | 17.55 (16.94 – 18.15) | 31.81 (31.55 – 32.07) |
| 2016 | 27.12 (24.23 – 30.01) | 14.21 (13.48 – 14.93) | 22.27 (21.63 – 22.90) | 17.61 (17.02 – 18.20) | 31.00 (30.75 – 31.25) |
| 2017 | 26.51 (23.74 – 29.28) | 14.50 (13.80 – 15.20) | 22.27 (21.65 – 22.89) | 17.84 (17.26 – 18.43) | 32.12 (31.87 – 32.38) |
| 2018 | 23.43 (20.94 – 25.93) | 14.92 (14.23 – 15.62) | 22.63 (22.01 – 23.24) | 18.19 (17.61 – 18.76) | 32.67 (32.42 – 32.92) |
| 2019 | 22.86 (20.43 – 25.29) | 14.41 (13.75 – 15.08) | 22.39 (21.79 – 22.99) | 18.47 (17.90 – 19.03) | 32.73 (32.48 – 32.98) |
| 2020 | 30.37 (27.64 – 33.11) | 16.04 (15.36 – 16.73) | 26.12 (25.48 – 26.76) | 22.16 (21.55 – 22.77) | 36.57 (36.31 – 36.84) |
| 2021 | 28.69 (26.00 – 31.60) | 16.83 (16.12 – 17.55) | 26.53 (25.87 – 27.19) | 22.16 (21.56 – 22.78) | 40.23 (39.94 – 40.52) |
| 2022 | 26.43 (23.93 – 29.13) | 15.85 (15.19 – 16.52) | 25.48 (24.85 – 26.12) | 20.42 (19.86 – 21.00) | 38.07 (37.80 – 38.34) |
| 2023 | 25.63 (23.24 – 28.22) | 14.67 (14.05 – 15.31) | 24.93 (24.31 – 25.56) | 18.84 (18.30 – 19.39) | 36.76 (36.49 – 37.03) |
| 2024 | 23.18 (20.98 – 25.58) | 14.09 (13.50 – 14.69) | 24.72 (24.12 – 25.33) | 17.94 (17.43 – 18.45) | 35.87 (35.61 – 36.13) |
| **Total** | **26.47**  **(23.28 – 29.69)** | **16.44**  **(15.49 – 17.39)** | **26.50**  **(25.76 – 27.25)** | **20.93**  **(20.16 – 21.70)** | **34.79**  **(34.52 – 35.07)** |

CI: Confidence Interval

**Supplemental Table 6** Ischemic heart disease and Cardiac Arrhythmia related Age Adjusted Mortality Rates per 100,000 Stratified by Census Region in the United States, 1999 to 2024

| **Age Adjusted Mortality Rates per 100,000 (95% CI)** | | | | |
| --- | --- | --- | --- | --- |
| **Year** | **Northeast** | **Midwest** | **South** | **West** |
| 1999 | 42.15  (41.49 – 42.80) | 45.53  (44.89 – 46.17) | 40.68  (40.18 – 41.19) | 39.75  (39.09 – 40.42) |
| 2000 | 38.94  (38.32 – 39.57) | 41.71  (41.10 – 42.32) | 36.35  (35.88 – 36.82) | 36.84  (36.21 – 37.48) |
| 2001 | 40.46  (39.82 – 41.09) | 41.15  (40.55 – 41.76) | 37.30  (36.83 – 37.78) | 38.31  (37.67 – 38.95) |
| 2002 | 38.97  (38.35 – 39.59) | 40.62  (40.02 – 41.21) | 36.49  (36.02 – 36.96) | 38.02  (37.39 – 38.65) |
| 2003 | 36.86  (36.26 – 37.46) | 38.51  (37.93 – 39.09) | 35.25  (34.79 – 35.71) | 36.15  (35.55 – 36.76) |
| 2004 | 34.25  (33.68 – 34.83) | 36.40  (35.84 – 36.96) | 31.90  (31.47 – 32.33) | 33.35  (32.77 – 33.93) |
| 2005 | 33.56  (32.99 – 34.12) | 37.00  (36.45 – 37.56) | 32.57  (32.14 – 33.00) | 33.20  (32.63 – 33.77) |
| 2006 | 30.59  (30.06 – 31.13) | 35.35  (34.81 – 35.90) | 30.35  (29.94 – 30.76) | 32.00  (31.45 – 32.55) |
| 2007 | 30.50  (29.97 – 31.03) | 34.17  (33.64 – 34.70) | 29.04  (28.64 – 29.43) | 30.06  (29.53 – 30.58) |
| 2008 | 30.25  (29.72 – 30.77) | 34.15  (33.62 – 34.67) | 28.34  (27.95 – 28.73) | 30.60  (30.07 – 31.12) |
| 2009 | 28.48  (27.97 – 28.99) | 32.48  (31.97 – 32.99) | 27.51  (27.13 – 27.89) | 28.73  (28.22 – 29.23) |
| 2010 | 29.53  (29.01 – 30.04) | 31.60  (31.10 – 32.10) | 27.48  (27.11 – 27.86) | 29.23  (28.72 – 29.73) |
| 2011 | 29.50  (28.99 – 30.01) | 32.10  (31.60 – 32.60) | 26.76  (26.40 – 27.12) | 29.54  (29.04 – 30.04) |
| 2012 | 28.92  (28.41 – 29.42) | 31.30  (30.81 – 31.79) | 27.10  (26.74 – 27.46) | 28.84  (28.35 – 29.32) |
| 2013 | 29.11  (28.61 – 29.61) | 31.47  (30.98 – 31.96) | 27.07  (26.72 – 27.43) | 28.86  (28.38 – 29.33) |
| 2014 | 28.71  (28.21 – 29.20) | 31.29  (30.80 – 31.77) | 27.26  (26.90 – 27.61) | 28.31  (27.84 – 28.77) |
| 2015 | 28.71  (28.22 – 29.20) | 32.08  (31.59 – 32.56) | 27.83  (27.48 – 28.18) | 29.33  (28.87 – 29.80) |
| 2016 | 27.56  (27.08 – 28.04) | 31.22  (30.74 – 31.70) | 27.00  (26.66 – 27.34) | 29.29  (28.83 – 29.75) |
| 2017 | 27.48  (27.00 – 27.95) | 32.67  (32.18 – 33.15) | 28.04  (27.70 – 28.39) | 29.85  (29.39 – 30.31) |
| 2018 | 27.85  (27.38 – 28.32) | 33.18  (32.70 – 33.66) | 28.86  (28.51 – 29.20) | 29.82  (29.37 – 30.27) |
| 2019 | 27.68  (27.21 – 28.14) | 32.88  (32.40 – 33.35) | 29.26  (28.92 – 29.61) | 29.43  (28.99 – 29.87) |
| 2020 | 31.93  (31.43 – 32.43) | 37.25  (36.75 – 37.76) | 33.07  (32.71 – 33.43) | 31.62  (31.17 – 32.07) |
| 2021 | 32.30  (31.80 – 32.81) | 40.15  (39.62 – 40.69) | 36.35  (35.97 – 36.74) | 34.75  (34.26 – 35.24) |
| 2022 | 30.75  (30.26 – 31.23) | 36.96  (36.46 – 37.46) | 34.53  (34.16 – 34.89) | 33.24  (32.78 – 33.70) |
| 2023 | 29.36  (28.89 – 29.84) | 35.34  (34.86 – 35.84) | 33.80  (33.45 – 34.16) | 31.29  (30.85 – 31.74) |
| 2024 | 27.83  (27.38 – 28.29) | 35.15  (34.67 – 35.63) | 32.94  (32.60 – 33.29) | 29.86  (29.44 – 30.29) |
| **Total** | **31.62**  **(31.10 – 32.15)** | **35.45**  **(34.93 – 35.97)** | **31.27**  **(30.88 – 31.67)** | **31.93**  **(31.42 – 32.45)** |
| CI: Confidence Interval | | | | |

**Supplemental Table 7** Ischemic heart disease and Cardiac Arrhythmia related Age Adjusted Mortality Rates per 100,000 Stratified by Urbanization in the United States, 1999 to 2020

|  | | |
| --- | --- | --- |
| **Age Adjusted Mortality Rates per 100,000 (95% CI)** | | |
| **Year** | **Metropolitan** | **Non-Metropolitan** |
| 1999 | 41.22 (40.89 – 41.56) | 45.24 (44.52 – 45.96) |
| 2000 | 37.41 (37.09 – 37.72) | 42.1844 (41.49 – 42.88) |
| 2001 | 38.42 (38.11 – 38.74) | 42.08 (41.39 – 42.77) |
| 2002 | 37.58 (37.27 – 37.89) | 41.70 (41.02 – 42.38) |
| 2003 | 35.59 (35.29 – 35.89) | 40.92 (40.25 – 41.59) |
| 2004 | 32.95 (32.66 – 33.24) | 37.47 (36.83 – 38.11) |
| 2005 | 33.08 (32.79 – 33.36) | 38.10 (37.45 – 38.74) |
| 2006 | 31.03 (30.76 – 31.30) | 35.99 (35.37 – 36.61) |
| 2007 | 29.78 (29.519 – 30.05) | 35.18 (34.57 – 35.79) |
| 2008 | 29.62 (29.36 – 29.89) | 34.97 (34.37 – 35.58) |
| 2009 | 28.12 (27.87 – 28.38) | 33.93 (33.34 – 34.52) |
| 2010 | 28.24 (27.99 – 28.49) | 34.06 (33.47 – 34.66) |
| 2011 | 28.30 (28.05 – 28.55) | 33.25 (32.67 – 33.83) |
| 2012 | 27.77 (27.53 – 28.01) | 33.91 (33.33 – 34.49) |
| 2013 | 27.95 (27.71 – 28.19) | 33.37 (32.80 – 33.94) |
| 2014 | 27.52 (27.29 – 27.75) | 34.47 (33.89 – 35.05) |
| 2015 | 28.16 (27.92 – 28.39) | 34.88 (34.31 – 35.46) |
| 2016 | 27.45 (27.2 – 27.69) | 34.01 (33.4 – 34.58) |
| 2017 | 28.18 (27.95 – 28.41) | 35.34 (34.77 – 35.92) |
| 2018 | 28.44 (28.21 – 28.67) | 36.80 (36.22 – 37.38) |
| 2019 | 28.29 (28.06 – 28.51) | 37.57 (36.99 – 38.15) |
| 2020 | 31.85 (31.61 – 32.09) | 41.64 (41.04 – 42.25) |
| **Total** | **31.23 (30.96 – 31.49)** | **37.14 (36.52 – 37.76)** |

CI: Confidence Interval

**Supplemental Table 8** Ischemic heart disease and Cardiac Arrhythmia related Age Adjusted Mortality Rate per 100,000 stratified by States in the United States, 1999-2024

|  | |
| --- | --- |
| **States** | **Age Adjusted Mortality Rate per 100,000 (95% CI)** |
| Alabama | 24.63 (24.07 – 25.20) |
| Alaska | 24.46 (22.59 – 26.43) |
| Arizona | 27.69 (27.20 – 28.19) |
| Arkansas | 33.96 (33.11 – 34.84) |
| California | 31.64 (31.41 – 31.87) |
| Colorado | 32.59 (31.91 – 33.28) |
| Connecticut | 22.82 (22.25 – 23.41) |
| Delaware | 40.79 (39.25 – 42.38) |
| District of Columbia | 25.28 (23.59 – 27.06) |
| Florida | 28.08 (27.82 – 28.34) |
| Georgia | 19.93 (19.55 – 20.31) |
| Hawaii | 22.16 (21.27 – 23.09) |
| Idaho | 36.08 (34.89 – 37.30) |
| Illinois | 25.73 (25.38 – 26.09) |
| Indiana | 39.23 (38.61 – 39.86) |
| Iowa | 37.99 (37.15 – 38.84) |
| Kansas | 34.40 (33.53 – 35.28) |
| Kentucky | 36.42 (35.69 – 37.17) |
| Louisiana | 30.71 (30.01 – 31.41) |
| Maine | 37.90 (36.69 – 39.14) |
| Maryland | 37.42 (36.78 – 38.07) |
| Massachusetts | 27.18 (26.70 – 27.67) |
| Michigan | 32.30 (31.86 – 32.75) |
| Minnesota | 38.78 (38.10 – 39.46) |
| Mississippi | 31.26 (30.39 – 32.14) |
| Missouri | 30.74 (30.20 – 31.29) |
| Montana | 28.54 (27.28 – 29.85) |
| Nebraska | 39.31 (38.15 – 40.49) |
| Nevada | 21.97 (21.22 – 22.74) |
| New Hampshire | 31.85 (30.71 – 33.04) |
| New Jersey | 29.97 (29.53 – 30.41) |
| New Mexico | 24.38 (23.52 – 25.26) |
| New York | 28.42 (28.13 – 28.72) |
| North Carolina | 34.17 (33.70 – 34.65) |
| North Dakota | 37.55 (35.84 – 39.32) |
| Ohio | 44.38 (43.91 – 44.86) |
| Oklahoma | 40.85 (40.01 – 41.711) |
| Oregon | 40.63 (39.83 – 41.43) |
| Pennsylvania | 35.72 (35.34 – 36.11) |
| Rhode Island | 44.70 (43.21 – 46.24) |
| South Carolina | 37.89 (37.19 – 38.59) |
| South Dakota | 39.59 (37.91 – 41.33) |
| Tennessee | 45.39 (44.73 – 46.07) |
| Texas | 34.44 (34.12 – 34.76) |
| Utah | 23.07 (22.22 – 23.94) |
| Vermont | 48.12 (46.11 – 50.21) |
| Virginia | 29.35 (28.85 – 29.84) |
| Washington | 41.59 (40.97 – 42.22) |
| West Virginia | 47.61 (46.41 – 48.85) |
| Wisconsin | 42.31 (41.64 – 43.00) |
| Wyoming | 35.90 (33.85 – 38.04) |
| CI: Confidence Interval | |

**Supplemental Table 9** Number of Ischemic heart disease and Cardiac Arrhythmia related Deaths, Place of Death in the United States, 1999-2024

| **Deaths** | | | | | | | | |
| --- | --- | --- | --- | --- | --- | --- | --- | --- |
| **Year** | **Medical Facility - Inpatient** | **Medical Facility - Outpatient or ER** | **Medical Facility - Dead on Arrival** | **Decedent's home** | **Hospice facility** | **Nursing home/long term care** | **Other** | **Place of death unknown** |
| 1999 | 29906 | 11081 | 1697 | 13317 | 0 | 15897 | 1588 | 17 |
| 2000 | 27469 | 9727 | 1414 | 12592 | 0 | 15066 | 1690 | 25 |
| 2001 | 28334 | 9806 | 1295 | 13165 | 0 | 16114 | 1847 | 13 |
| 2002 | 27628 | 9539 | 1239 | 13469 | 0 | 16190 | 2076 | 0 |
| 2003 | 26793 | 9086 | 1014 | 13451 | 71 | 15680 | 2140 | 164 |
| 2004 | 24261 | 8564 | 926 | 13181 | 143 | 14719 | 2121 | 188 |
| 2005 | 24603 | 8524 | 902 | 13596 | 499 | 15516 | 2001 | 172 |
| 2006 | 23593 | 8032 | 868 | 13293 | 615 | 14747 | 1873 | 183 |
| 2007 | 22904 | 7674 | 814 | 13329 | 914 | 14449 | 1933 | 112 |
| 2008 | 23298 | 7429 | 702 | 13597 | 1149 | 14272 | 2031 | 642 |
| 2009 | 21526 | 7456 | 640 | 13684 | 1172 | 13750 | 2080 | 1000 |
| 2010 | 21806 | 7635 | 676 | 14455 | 1625 | 14078 | 2250 | 59 |
| 2011 | 22205 | 7699 | 640 | 14976 | 1869 | 14408 | 2365 | 41 |
| 2012 | 21592 | 7569 | 585 | 15889 | 2193 | 14607 | 2592 | 41 |
| 2013 | 21640 | 7737 | 580 | 16857 | 2338 | 14918 | 2740 | 26 |
| 2014 | 21768 | 7813 | 514 | 17771 | 2547 | 14945 | 2494 | 44 |
| 2015 | 22817 | 7668 | 465 | 18810 | 2973 | 15511 | 2662 | 19 |
| 2016 | 22771 | 7548 | 398 | 19210 | 3204 | 14825 | 2653 | 0 |
| 2017 | 23710 | 7503 | 361 | 20765 | 3650 | 15385 | 2949 | 11 |
| 2018 | 24506 | 7642 | 318 | 22154 | 3858 | 15744 | 3039 | 10 |
| 2019 | 24189 | 7980 | 321 | 23083 | 4415 | 15618 | 3247 | 14 |
| 2020 | 27795 | 8386 | 276 | 29063 | 4524 | 16017 | 3961 | 21 |
| 2021 | 30437 | 8340 | 285 | 30342 | 4958 | 14695 | 4091 | 14 |
| 2022 | 29598 | 8521 | 259 | 30069 | 5027 | 15348 | 4303 | 0 |
| 2023 | 27330 | 8006 | 257 | 28707 | 5461 | 15892 | 3779 | 0 |
| 2024 | 27499 | 8081 | 237 | 29266 | 5555 | 15639 | 3711 | 11 |
| **Total** | **649,978** | **215,046** | **17,683** | **478,091** | **58,760** | **394,030** | **68,216** | **2,827** |
